# The effectiveness and cost-effectiveness of attachment-based family therapy for young adults with high suicidal ideation: protocol of a randomized controlled trial

**DOI:** 10.1186/s13063-024-08499-7

**Published:** 2024-10-16

**Authors:** Claudi Bockting, Guy Bosmans, Nele Bergers, Luana Gavan, Mickaël Hiligsmann, Derek de Beurs, Geert Molenberghs, Ben Wijnen, Joran Lokkerbol, Nadia van der Spek

**Affiliations:** 1 Department of Psychiatry, Amsterdam University Medical Centre, Location AMC, Amsterdam, North Holland 1105 AZ The Netherlands; 2 Institute of Advanced Study, Centre for Urban Mental Health, University of Amsterdam, Amsterdam, The Netherlands; 3 KU Leuven, Louvain, Flanders Belgium; 4 Faculty of Health Medicine and Life Sciences, Maastricht University, Maastricht, Limburg The Netherlands; 5 University of Amsterdam, Amsterdam, Noord-Holland The Netherlands; 6 Hasselt University, Hasselt, Limburg, Belgium; 7 Center of Economic Evaluation, Trimbos-Instituut, Utrecht, The Netherlands; 8 Mental Health and Addiction, Trimbos-Instituut, Utrecht, The Netherlands; 9 Mental Health Care Centre De Amsterdamse, Amsterdam, The Netherlands; 10 Academic Workplace Shift Left, Arkin Mental Health Centre, Amsterdam, The Netherlands

**Keywords:** Young adult, Suicide prevention, Suicidal ideation, Suicidality, Attachment-based family therapy, Randomized controlled trial, Cost-effectiveness, Psychotherapy

## Abstract

**Background:**

Young adult suicidality is worldwide a prevalent mental health problem and the number one cause of death, with devastating consequences for individuals and their families, and substantial economic costs. However, psychological and pharmacological treatments currently recommended in guidelines for treatment of high-risk youth for fatal suicide have limited effect. In line with the World Health Organization’s (WHO) recommendation to involve the family in treatment of these youth, attachment-based family therapy (ABFT) was developed, a 16-week attachment and emotion-focused treatment, implemented in mental health care settings across various European countries in the past years, and becoming increasingly popular among therapists. However, the (cost-)effectiveness of ABFT has not been studied in emerging adults. In the proposed pragmatic randomized controlled trial (RCT), we aim to evaluate the effectiveness and cost-effectiveness of ABFT compared to treatment as usual (TAU) on suicidality, as delivered in daily practice.

**Methods:**

This pragmatic multicenter study in the Netherlands and Belgium includes 13 participating sites. Participants are suicidal young adults (≥ 31 SIQ-JR score) between 16 and 30 years old who seek mental health treatment (*n* = 142) and their caregivers. The primary outcome is suicidality (SIQ-JR), with assessments at baseline, post-intervention (5 months after baseline), 3, 6, and 12 months after intervention. We predict that, compared to TAU, ABFT will lead to a stronger reduction in suicidality and will be more cost-effective, over the course of all time points. We also expect stronger decreases in depressive symptoms, given that suicidality is very common in individuals with depressive disorder, as well as more improvement in family functioning, autonomy, entrapment, and young adult attachment, in the ABFT condition.

**Discussion:**

This study can contribute to improving the care for suicidal youngsters with high mortality risk. Treatment of suicidal emerging adults is understudied. The results will inform clinical guidelines and policy makers and improve treatment of suicidal emerging adults.

**Trial registration:**

This trial is registered on ClinicalTrials.gov (NCT05965622, first posted on July 28, 2023).

**Supplementary Information:**

The online version contains supplementary material available at 10.1186/s13063-024-08499-7.

## Introduction

### Background and rationale {6a}

Emerging adult suicide is a serious problem around the world. Suicide is estimated to be the fourth cause of death in the 15–29 years age group worldwide [1] and the first cause of death in that age group in the Netherlands [2]. Moreover, for each fatal suicide, approximately 20 non-fatal suicide attempts have been registered [3]. Identifying and treating ultra-high suicide risk youth is one of the pressing current challenges in societies across the world [4]. The emotional cost for families and loved ones cannot be measured, but is clearly devastating. In addition, the economic and societal cost of fatal suicide is very high and includes medical costs for individuals and families, lost income for families, and lost productivity for victims and family members [5, 6]. Suicidal ideation is very prevalent among people with mental health disorders, such as depression [7]. Suicidal ideation can result in suicide attempts and fatal suicide, but is in itself also very burdensome for individuals [8, 9]. The societal and economic burden of patients with suicidal ideation is higher compared to depressed patients without suicidal ideation [10]. Therefore, suicidal ideation needs to be targeted with treatment. However, research on the treatment of youth suicidality lags significantly behind. Current treatments, including pharmacological treatment of suicidal emerging adults, have limited effects [11, 12]. Treatment programs that can successfully treat emerging adults at high suicide risk are greatly needed and the societal impact of providing cost-effective treatment could be significant.

Thus, tackling psychological mechanisms in treatment that contribute to decreasing young adult suicidality and depression is a critical next step to tackle suicidality. Until recently, standard treatment of suicidality consisted of cognitive behavioral therapy (CBT), dialectical behavioral therapy (DBT), and/or medication, but all showed limited effects on suicidality [11, 13, 14]. Even worse, a recent meta-analysis of 50 years of treatment research found that the effect of existing treatments only produces small effects on suicidal behavior [11]. A recent review [15] shows that there is only a limited number of evidence-based treatment options for adolescents that all only have limited treatment effects on suicidality, calling for more trials specifically targeting suicidality. Mixed support for the current treatments has contributed to the general recommendation to provide psychological treatments aimed at improving family relationships [3, 16].

Family therapy is a less explored treatment option for suicidality. There is increasing evidence that points at the importance of working with families and addressing the unmet need for belonging and perceived burdensomeness when targeting youth suicidality [15–19]. Even for emerging adults, who are in a developmental phase of striving for autonomy, not addressing family conflict might exacerbate the problem or inhibit the family from being a source of support for the patient [20]. In most cases, these emerging adults will either be living at home or be dependent on their families. The WHO recommends to involve the family in the treatment of suicidal youth as a powerful way to treat suicidality [3]. One program that has been developed to specifically target suicidal youth, and that specifically focuses on the family, is attachment-based family therapy (ABFT) [21–23].

ABFT is a promising intervention for adolescents, gaining popularity around the world, that involves both the youngster and the caregivers. ABFT uses family therapeutic techniques to restore attachment relationships between members as a strategy to decrease youngsters’ levels of depression and suicide risk [22]. Multiple studies have shown high acceptability and feasibility of ABFT for both adolescents and emerging adults [20, 24–27]. However, effectiveness studies have shown mixed results on suicidal ideation [21, 23, 28, 29]. Moreover, a recent meta-analysis on the RCTs up until now has shown no effect of ABFT on suicidal ideation among adolescents (Schulte-Frankenfeld PM, Breedvelt JF, Brouwer ME, van der Spek N, Bosmans G, Bockting CL: Effectiveness of attachment-based family therapy for suicidal adolescents and young adults: a systematic review and meta-analysis, forthcoming). ABFT has never been studied in an RCT among emerging adults, who are in a developmental phase characterized by a different type of parent–child relationship. Therefore, more research is needed to establish whether ABFT is a cost-effective treatment for suicidality and to investigate its effectiveness for emerging adults, instead of adolescents.

## Objectives {7}

The current RCT will compare ABFT added to treatment as usual (ABFT + TAU) to TAU alone among emerging adults between 16 and 30 years old, with high levels of suicidality, receiving care in mental health care facilities in the Netherlands and Belgium. The main goal of the study is to assess the effectiveness and cost-effectiveness of ABFT added to TAU, compared to TAU alone, on suicidality in emerging adults over the course of five time points (baseline, post-treatment, 3-, 6-, and 12-month follow-up). Secondly, the effects of ABFT on depression, self-injury, entrapment, autonomy, family functioning, and young adult attachment will be investigated. We hypothesize that ABFT + TAU will be more effective than TAU alone in decreasing suicidality, while also being more cost-effective.

## Trial design {8}

This study is a randomized controlled pragmatic multicenter trial comparing attachment-based family therapy (ABFT) added on to treatment as usual (TAU) with TAU. It is a parallel group, two-arm, superiority trial with a 1:1 allocation.

## Methods: participants, interventions, and outcomes

### Study setting {9}

Participants are suicidal emerging adults between 16 and 30 years old, who seek treatment, and their parents or caregivers. The study will take place in 12 mental health care centers in the Netherlands and Belgium where suicidal patients are treated, including both inpatient and outpatient clinics, academic and non-academic treatment centers, and private practices. A list of all participating study sites can be found in the Supplementary Material.

### Eligibility criteria {10}

#### Participating sites and therapists

Study sites are eligible to participate if they are located in either the Netherlands or Belgium, have sufficient ABFT-trained therapists, have been trained in the study protocol, have signed a clinical trial agreement with the sponsor, and if they have been approved for participation by the Medical Research Ethics Committee (MREC). Therapists eligible to deliver the study intervention are licensed clinical psychologists, psychotherapists, or psychiatrists who received at least 9 days of training and 20 h of supervision in ABFT.

#### Study participants

After signing informed consent, the eligibility of patients will be assessed in a pretreatment session, using the Suicidal Ideation Questionnaire-JR (SIQ-JR), the Structured Clinical Interview for DSM-5 (SCID-5-S), and patient records. Inclusion criteria are as follows: (a) between 16 and 30 years old, (b) a score above the cut-off of the SIQ-JR monthly ≥ 31, and (c) at least one parent or primary caregiver that participates in the assessments and treatment. This could be a biological parent, stepparent, grandparent, other relative, or a foster parent. Exclusion criteria are as follows: (a) other DSM-5 disorders: severe alcohol or cannabis use disorder, for all other substances: moderate or severe substance use disorder, conduct disorder, evidence of psychotic features, or prior psychosis; (b) severe cognitive impairment (e.g., mental retardation, severe developmental disorders) as evidenced by educational records, parental report, and/or clinical impression; and (c) other circumstances that might affect participation (e.g., severe medical disorder, relocation). The exclusion criteria relating to substance use disorder and psychotic features will be assessed using the SCID-5-S.

### Who will take informed consent? {26a}

After regular intake procedures in the treatment facility, patients with suicidality and their caregiver(s) will be informed about the study and asked to participate by a local researcher. They will receive both oral and written information on the study and have an opportunity to ask any questions. After at least 1 week, the patients who are willing to participate will meet with a local researcher who will obtain written consent from both the patient and their parent(s) or caregiver(s). The local researcher has been trained in the study protocol.

### Additional consent provisions for collection and use of participant data and biological specimens {26b}

Not applicable, as there are no ancillary studies related to the current study.

## Interventions

### Explanation for the choice of comparators {6b}

TAU was chosen as a comparator for the ABFT + TAU condition as a way of distinguishing the effectiveness of ABFT added to TAU, in the intervention group as compared to TAU alone. As the study population is highly suicidal young adults, it was deemed unethical to assign participants in the comparator condition to a non-active control condition such as waitlist-control. TAU can be any psychological or psychopharmacological intervention except for family therapy, which allows therapists at the participating study sites to choose an appropriate treatment plan for the TAU participants.

### Intervention description {11a}

Attachment-based family therapy (ABFT) [30] is a manualized treatment including 16 sessions that emerges from interpersonal theories that suggest suicide can be precipitated, exacerbated, or buffered against by the quality of family relationships. ABFT is delivered by licensed clinical psychologists, psychotherapists, or psychiatrists who received 9 days of training and 20 h of supervision in ABFT.

Although ABFT therapists implement behavior-focused and psychoeducational interventions, the model is primarily a process-oriented, emotion-focused treatment, guided by a semi-structured treatment protocol. Suicide risk is monitored in every session by the therapist. Treatment is organized around five specific tasks, each with a distinct process and goal. Each task refers to a specific treatment goal and offers a principle-driven guideline to achieve each goal. Task 1 (relational reframing) focuses on shifting the family’s treatment goal from “fixing the young adult” to improving family relationships. The task 1 session includes a discussion of what prohibits the youngster from turning to his or her parent(s)/caregiver(s) for help when feeling so hopeless and distressed that they contemplate suicide. Family barriers range from stress due to the youngster’s symptoms, history of negative interactions and communication, abuse, neglect, abandonment, and/or parental psychopathology. Even well-functioning families need help effectively managing youth’s suicidality. Task 2 (alliance building with the youngster, three to four sessions) aims to help the youngster gain a better understanding of how ruptures in trust with the parents contribute to conflict and emotional distance. Emerging adults are then motivated and prepared to discuss these ruptures with their parents. Task 3 (alliance with the parents, three to four sessions) aims to help the parents become more empathic and sensitive to the youngster’s emotional needs. Task 4 (the attachment task, three to four sessions) brings all the family members back together to discuss some of the issues and ruptures that were identified in tasks 2 and 3. ABFT considers caregiver support in response to the youngster’s shared experiences as a corrective attachment experience that helps repair trust. Task 5 (promoting autonomy) assumes that task 4 has built up enough trust that the parents can once again serve as a secure base to support the young adult’s competency and autonomy development. These five tasks provide a scaffold to guide the treatment.

#### Treatment as usual (TAU)

Participants in both arms will receive TAU; in the experimental condition ABFT will be delivered as an add on. Most treatment centers’ clinical practices rely heavily on the use of antidepressants and/or cognitive behavioral therapy (CBT) or dialectical behavior therapy (DBT). All regular interventions are allowed in TAU, except for systemic family therapy of more than 4 sessions in the first 5 months of the study (i.e., a family therapy session is defined as a contact of 30 min or longer with a family member or caregiver present). Parental involvement is generally part of treatment as usual and can comprise for instance psychoeducation, parental support, or skill training. We will monitor what TAU entails. “TAU alone” is compared to ABFT added to TAU.

### Criteria for discontinuing or modifying allocated interventions {11b}

For this trial, we made a few adjustments to the treatment manual. First, at the beginning of the treatment, a family-oriented safety plan will be made for all patients receiving ABFT and will be used at all times during treatment when the patient is at high risk. Second, the developmental task of emerging adults, which involves obtaining autonomy and launching into adulthood, is central in the treatment. For instance, the task 1 session is not mandatory if the young adult prefers to start with separate sessions. Task 4 is still about discussing ruptures, but also focuses on self-expression and individuating from the parents. No other treatment modifications will be performed. The possibility of discontinuing the allocated intervention will be discussed within each study site and with the participants’ main therapist, taking into account participants’ wishes and any safety-related concerns.

### Strategies to improve adherence to interventions {11c}

Therapists will receive supervision over their cases in the study. All ABFT sessions will be videotaped and a randomly selected 15% of the tapes will be scored by an independent rater to score treatment fidelity. Treatment fidelity will be assessed with the Therapist Behavior Rating Scale-3 (TBRS-3; [31]). The TBRS-3 items demonstrated reliability with ICCs ranging from good (0.69) to excellent (0.96).

### Relevant concomitant care permitted or prohibited during the trial {11d}

Any concomitant care is permitted during the trial, except for more than 4 sessions of systemic family therapy during the first 5 months, for participants in the TAU treatment condition. For the purpose of this trial, we define family therapy as any contact of 30 min or longer with a family member or caregiver present.

### Provisions for post-trial care {30}

This is not applicable, as participants in the study can always benefit from ancillary or post-trial care through their regular health care provider and health insurance. Although there are no expected risks for the participants, the sponsor has a liability insurance in case of any harm arising to participants from participation in the study.

### Outcomes {12}

Socio-demographic factors and participant characteristics and childhood trauma using the Childhood Trauma Questionnaire (CTQ; [32]) will be measured at baseline only. The CTQ is a self-report 28-item questionnaire that measures 5 types of maltreatment. This questionnaire is not time-specific. Sum scores are calculated per subscale (range: 5–25); the subscales and cut-offs are as follows: emotional abuse (> 13), physical abuse (> 10), sexual abuse (> 8), emotional neglect (> 15), and physical neglect (> 10) [32]. We include this questionnaire at baseline only, since childhood trauma is an important predictor of suicidality and depression. In line with CONSORT 2010, differences between group with regard to demographics at baseline will be examined in consideration of the prognostic strength of the variables measured and the size of any chance imbalances that have occurred. In case of prognostically relevant imbalances, adjustments to the analyses will be made accordingly. In addition, differences between treatment groups on childhood trauma (i.e., CTQ) will be investigated and post hoc analyses will be done to examine whether childhood trauma could bias the effect between treatment group (and its interaction with time) and suicidality.

#### Primary outcome

Change in suicidal ideation and behavior (i.e., sum score, range: 0–90) as assessed by the Suicidal Ideation Questionnaire Junior (SIQ-JR; [33]) in the young adult from baseline to post-intervention, 3-, 6-, and 12-month follow-up. The time point of primary importance is the post-intervention assessment. The SIQ-JR is a 15-item questionnaire which assesses suicidal ideation in the past month and which has been used in many clinical studies. It forms a continuum ranging from thoughts of death and wanting to be dead, general and specific suicidal plans, preparations for/and actual suicide attempts. The measure was found to be internally consistent (*α* = 0.94) with a test–retest reliability of 0.89 over 3 weeks. Participants indicate the frequency of certain ideations and behaviors in the past month and respond to items like “I have thought about killing myself” on a 7-point Likert scale (0 not in the past month, till 6 almost every day in the past month). An extra item was added to assess suicide attempts (“I have tried to kill myself in the past month” (yes/no)). In case of a (fatal) suicide attempt, the highest SIQ score will be given (90) at that time point. This score will be given based on additional clinical assessment (e.g., to discern between suicide attempt as behavior with intention to die and non-suicidal self-injurious behavior).

#### Secondary outcomes

**Fig. 1:**
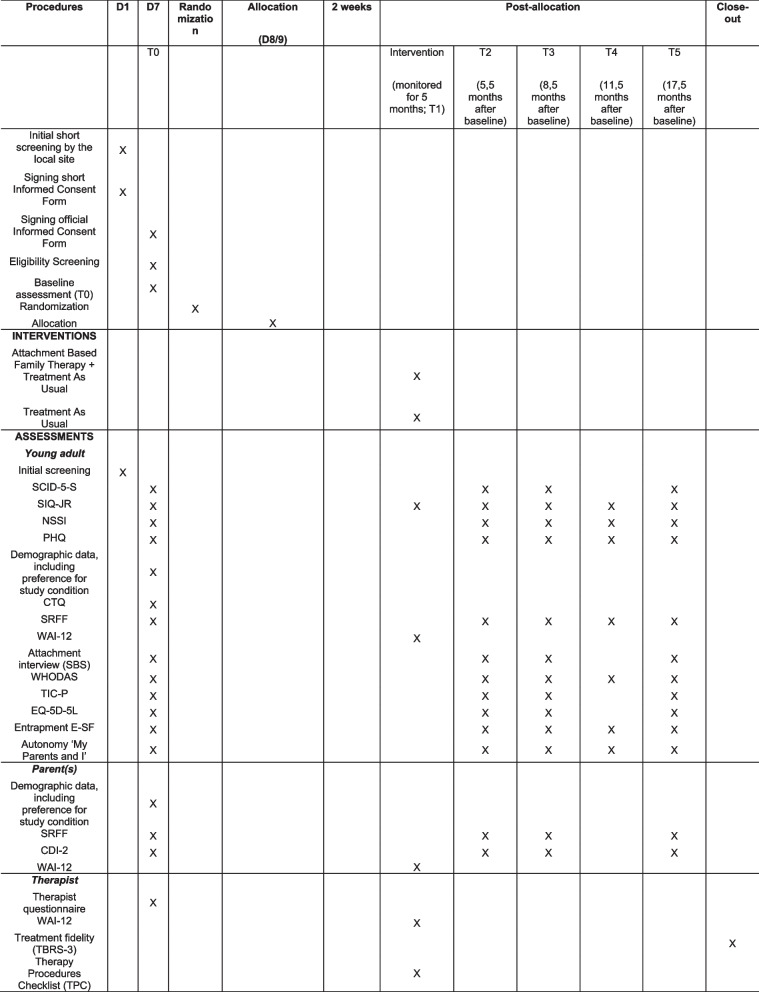
SPIRIT figure

All secondary outcomes are assessed at baseline, post-intervention, 3-month, and 12-month follow-up. In addition, several outcomes are also assessed at 6-month follow-up (see Fig. 1 for an overview per outcome measurement). Secondary outcomes are the individual change from baseline to the follow-up assessments in either sum scores or means, with the post-intervention measurement being the outcome of primary importance. The following measures will be used.

Suicide attempts and suicidality will be additionally investigated using the Structured Clinical Interview DSM 5 (SCID-5-S; [34]). The SCID-5-S is designed as a semi-structured diagnostic interview for making the major DSM-5 diagnosis. Validation and reliability studies have been done on the SCID-S versions DSM-V, DSM-IV, and DSM-III-R [35–37]. The results of these studies show that the validity and reliability of the SCID-S was good. The SCID-5-S can be administered effectively (mean 18.7 ± 11.6 min, median 15 min). Clinicians can use it after a brief training session. At all time points, current suicidal ideation, suicidal plans, and suicide attempt will be scored as either present or absent and change from the baseline to the follow-up assessments will be investigated.

Entrapment will be assessed using the Entrapment Short-Form Scale (E-SF; [38]). The E-SF is a 4-item questionnaire, which will be administered for the young adult only. Respondents are asked to respond to statements on internal and external entrapment on a 5-point scale, following which change in individual sum scores are calculated. Correlations between the sum of these four items and the 16-item full Entrapment Scale [39] were nearly perfect (0.94 for the clinical sample and 0.97 for the population-based sample).

Non-Suicidal Self-Injury (NSSI; [40]). This is a self-report 5-item questionnaire that assesses current and lifetime NSSI thoughts and behaviors and will be administered for the young adult only. No sum score can be calculated. NSSI thoughts and NSSI behaviors will be scored as present or absent at a specific time point.

Depressive symptomatology as assessed by Patient Health Questionnaire (PHQ-9; [41]). The PHQ-9 is recommended in the core outcome set for depression and represents the outcomes that matter most to patients with depression. It is a 9-item questionnaire, demonstrating good reliability and responsiveness to change, with Cronbach’s alphas ranging from 0.82 to 0.92 [42], and will be administered to the young adult only. Change in individual sum scores will be calculated and compared across time points (range: 0–27, with higher numbers indicating more depressive symptoms).

Major depression disorder diagnosis as assessed with the SCID-5-S (see description above) in the young adult only. A single variable will be created to reflect whether a diagnosis of depression has been given at that specific time point. Change from baseline to the follow-up assessment will be investigated.

World Health Organization Disability Assessment 2.0 (WHODAS 2.0; [43]) is part of the core outcome set for depression, consisting of 12 items covering the domains of cognition, mobility, self-care, getting along, life activities, and participation. The WHODAS 2.0 is a questionnaire with good validity and reliability [43] and will be administered to the young adult only. Change in individual sum scores will be calculated and compared across time points (range: 0–48, with higher numbers indicating more disability).

The Self Report Measures of Family Functioning (SRFF; [44]) is composed of 15 five-item factors measuring family functioning (e.g., cohesion, conflict, and democratic family style) with Cronbach’s alphas ranging from 0.63 to 0.91, with most in the 0.70 to 0.85 range. Analyses have yielded highly reliable and stable factors [44]. We will administer the SRFF to the young adult as well as the parents. Change in individual mean scores will be calculated per factor and compared across time points (range: 1–4, with higher numbers reflecting better family functioning).

Young adult attachment: To measure secure attachment in the young adult, we will use the secure base script task (SBS; [45]). This is a narrative task during which participants need to construct stories (six items for six stories) with prompt words that loosely suggest a secure base script, due to participants who know the script cannot suppress telling secure base script stories. The stories are then coded by trained researchers using a 7-point scale and an average individual change score will be computed, with higher scores indicating a more secure attachment. This measure correlates highly and significantly (*r* = 0.50) with attachment interviews that are considered valid in the field of attachment research [46, 47].

Autonomy of the young adult, as assessed by the 15-item questionnaire My Parents and I [48], a combination of items of the Emotional Autonomy Scale (EAS; [49]) and the Psychological Separation Inventory (PSI; 50), will be administered for the young adult only. The reliability of the combined scales, indicated by Cronbach’s alpha, amounts to 0.84. Mean change scores will be calculated and compared across time points (range: 1–5, with higher numbers indicating less autonomy).

Children’s Depression Inventory second edition (CDI-2, [51]) will be used to assess change of parental vision on the young adult’s depressive symptoms. The CDI-2 for parents consists of 17 items, scored on a 4-point Likert scale to indicate how often in the past 2 weeks symptoms occurred in their child. They respond to statements like “Looks sad” (0 = not at all, 1 = sometimes, 2 = often, 3 = almost always). A CDI-2 cut-off score of 16 is indicative of “significant” depressive symptoms according to the Dutch Mental Health Care guideline [52]. Mean change scores will be calculated and compared across time points.

#### Cost-effectiveness

Health care and associated costs and costs from productivity loss (TIC-P; [53]). The TIC-P is a questionnaire assessing participants’ healthcare resource use and productivity losses, including school and work dropout. The TIC-P will be administered at every measurement point and will inquire about healthcare resource use and productivity losses since the previous measurement (and in case of baseline for the 3 months prior to baseline). The TIC-P will be administered to the emerging adults only.

Health-related quality of life (EQ-5D-5L; [54, 55]). The EuroQOL five dimensions (EQ-5D) is a short questionnaire used to assess utilities of the young adult at each measurement. Utilities are converted into quality-adjusted life years (QALYs), using Dutch and Belgian tariffs.

Additional information is described in the statistical analysis section.

#### During-treatment assessment every 2 months

Working Alliance Inventory (12-item WAI; [56]). The WAI measures overall therapeutic alliance and consists of subscales measuring (quality of therapy) tasks, goals, and bonds. This measure has strong psychometric properties and has been used widely in psychotherapy research [57]. This measure will be administered to the young adult, the parent(s) or caregiver(s), and the therapist. Sum scores will be calculated and differences between treatment groups on working alliance (i.e., WAI scores) will be investigated. Next, post hoc analyses will be done to examine whether therapeutic alliance could bias the effect between treatment group (and its interaction with time) and suicidality (i.e., SIQ-JR).

Therapy Procedure Checklist (TPC; [58]). The TPC is developed to assess therapists’ reports of the techniques they employ when working with child and adolescent clients and will be used to monitor TAU in both study arms. TPC items encompass the three most common therapeutic models for youth: psychodynamic, cognitive, and behavioral. TPC scales have good internal consistency (all alpha > 0.86) and test–retest reliability (all *r* > 0.79) across samples.

### Participant timeline {13}

**Fig. 2:**
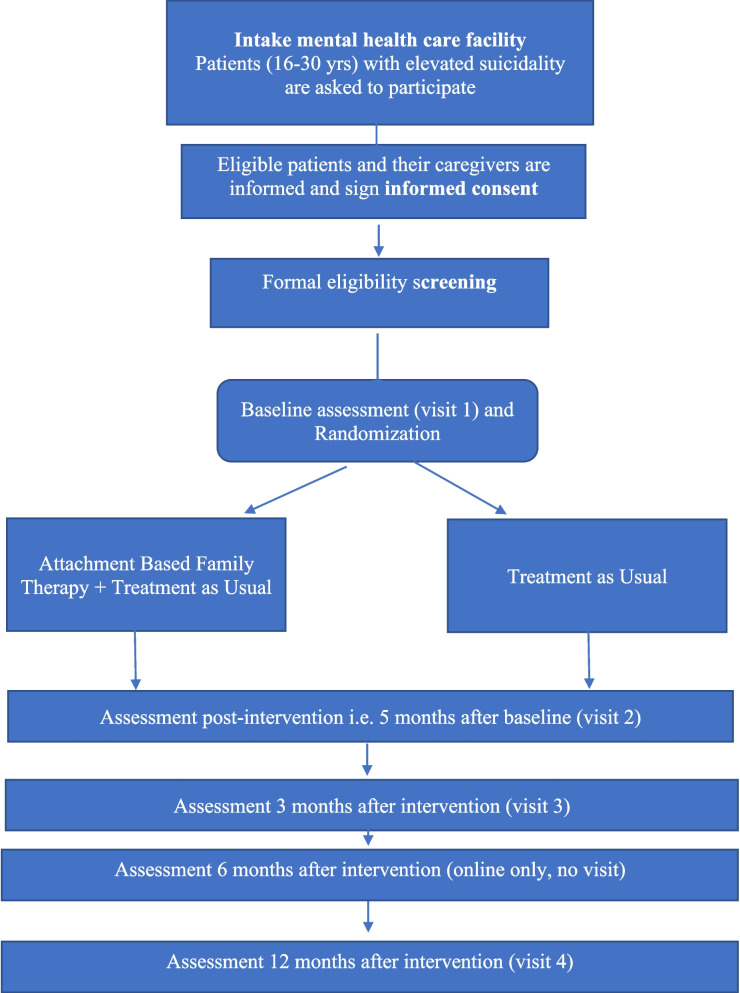
Flow chart of study procedure

Eligible patients and their parents/caregivers will be included in the study following the eligibility procedures previously described. After completing the baseline assessments, they will be randomized to ABFT + TAU or TAU alone. Follow-up interview assessments will take place post-intervention, at 3, and at 12 months, by a researcher blinded to the treatment condition. Questionnaire assessments are administered at baseline, post-intervention, and at 3, 6 (brief assessment for young adult only), and 12 months after intervention. Suicide attempts are registered during the interview assessments and in the questionnaires for the youngster. As long as the participant is in treatment at the site, suicide attempts will be reported by the therapist. For more detailed information, see Fig. 2.

### Sample size {14}

A recent meta-analysis, including three RCTs on ABFT for suicidal patients, found a Cohen’s *d* of 0.4 (Schulte-Frankenfeld PM, Breedvelt JF, Brouwer ME, van der Spek N, Bosmans G, Bockting CL: Effectiveness of attachment-based family therapy for suicidal adolescents and young adults: a systematic review and meta-analysis, forthcoming). Based on this estimated effect size (Cohen’s *d* = 0.4), focused on the primary outcome (suicidality) and the proposed statistical analyses (i.e., linear mixed modeling with five complete assessment time points), a base correlation across the 5 measurement of 0.5, an interclass correlation of patients “nested” in clinicians of 0.05, and assuming a design effect of 1.24 (given the clustered design), we will be needing ≈46 participants per arm for a power of 80% and a nominal type I error of 0.05 to be able to show a medium effect of ABFT. Based on previous trials [21, 23], we estimate a 35% dropout rate. We will therefore need a sample size of 71 (46/(1 − 0.35)) respondents per arm, resulting in a total of 142 participants. The power calculation was done using STATA sampsi package [59].

### Recruitment {15}

Participants will be recruited from the participating study sites by approaching them after intake or by searching for potential candidates in the existing caseload of therapists. Staff at the participating study sites have been informed about the study and can refer patients to the local study coordinator. The study will also be promoted via other channels such as personal network, news outlets, contact with suicide prevention organizations and general practitioners, as well as presence at relevant congresses and conferences, as a way to increase awareness about the study and reach the target sample size.

## Assignment of interventions: allocation

### Sequence generation {16a}

Eligible patients that gave informed consent, who are included in the study, will be randomized after baseline, through block randomization. A computer-generated randomization table with random block sizes will be prepared by an independent researcher not involved in the study. We will stratify on one variable: the number of previous suicide attempts (0 or > 0). Stratification will be done per country, in order to maintain balanced treatment conditions across both countries.

### Concealment mechanism {16b}

Randomization will be performed in Castor electronic data capture (Castor EDC) by an independent researcher not involved in the study. A participant will only be randomized after completing the baseline assessment. To conceal the allocation sequence, the randomization results are communicated to the local principal investigator on a “per enrolment” basis.

### Implementation {16c}

The result of the randomization procedure will be sent to the main study coordinator, who will then communicate it to the local principal investigator at the study site. The local principal investigator, who has been trained in the study protocol, is responsible for assigning participants to the correct therapist on the basis of the randomization outcome.

## Assignment of interventions: blinding

### Who will be blinded {17a}

The researcher performing the follow-up interview assessments will be blinded to the treatment condition. This researcher is not involved in the randomization procedure or in communicating the treatment allocation to local principal investigators. In addition, the independent researcher performing the statistical analyses will also be blinded, by receiving the necessary data files from which any information on the coding of the treatment condition has been removed.

### Procedure for unblinding if needed {17b}

Should unblinding occur inadvertently, then a different researcher will be assigned to perform follow-up assessments for that specific participant or to perform the statistical analyses. Cases of unblinding during the interview assessment (e.g., the participant mentions their treatment condition to the interviewer) will be documented in a protocol deviation log. There are no anticipated circumstances in which it would be deemed necessary to unblind either the follow-up assessor or the statistician.

## Data collection and management

### Plans for assessment and collection of outcomes {18a}

Data will be collected through questionnaires (using the clinical research platform Castor EDC) or (clinical) interviews. All data will be collected by researchers either from the study site or the sponsor, who have been trained in the study protocol, procedures, and assessments. An electronic case report form (eCRF; Castor EDC) is used to collect a part of the data; a copy of the blank CRF pages is kept as back-up. Some data will be collected through a CRF on paper and will be entered in Castor EDC afterwards. Access to the data collection system is based on individual login and access rights are only given to authorized staff. For each study-specific data collection, source documentation is available. Budget is allocated both for storage during the study as well as for archiving upon completion of the study. Budget is allocated for data management activities and creating a FAIR dataset (i.e., findable, accessible, interoperable, and reusable).

### Plans to promote participant retention and complete follow-up {18b}

Participant retention will be promoted by sending regular reminders for completion of assessments both by researchers and by the study therapists. Participants are also encouraged to complete all follow-up assessments by receiving a monetary incentive. To reduce the burden of follow-up assessments, study researchers will follow participants’ preference when planning an interview (e.g., face-to-face or online). To ensure complete follow-up assessments, participants will be given time at the end of a follow-up interview to complete the questionnaires corresponding to that visit.

Participants who decide to discontinue treatment can continue to participate in the study and complete any follow-up assessments according to the assessment schedule. If participants decide to discontinue study participation, they can continue treatment without any additional consequences. An early termination assessment will be done for participants who decide to discontinue participation. This assessment will inquire about their reasons for discontinuing participation and their experience with participation in the trial.

### Data management {19}

Data entry will only be done by trained researchers from the sponsor team (for the interview data) and directly by the participants (for the questionnaire data). All data entered in the eCRF will be checked against the paper CRF by a researcher independent from the researcher who initially entered the data. The data will be stored in a generic and machine actionable format, i.e., SPSS, R, or excel. The acquired data will be stored as read-only file and a new file will be created for further processing and statistical analysis. All data processing and analysis will be programmed in syntax or script files. Descriptive comments will be added to the syntax or script files. Data sets and syntax or script files will be placed under version control. Data corrections in this phase will be programmed in syntax or script files. With regard to sharing data for processing or statistical analysis, data will be transferred in a secure way. Encoded data from participating sites will be collected using secured links or during a site visit.

### Confidentiality {27}

All study and medical data will be coded and handled confidentially in accordance with the European Union General Data Protection Regulation and the Dutch Act on Implementation of the General Data Protection Regulations. Participant data will be coded with a unique participant number and stored in Castor EDC. A subject identification code list linking these participant numbers to the participant identification data will be created. This code list will only be available to the principal investigator of the study, staff members authorized by the principal investigator, the study monitoring agency, and the Health and Youth Care Inspectorate. Statistical experts involved in the study who will analyze the (cost-)effectiveness of the intervention will only receive the coded data. Original source documents will be archived for a period of 15 years after the study report has been finalized and will be thereafter destroyed. A Data Protection Impact Assessment (DPIA) has been performed. An informed consent procedure has been set up that describes the data set, time span of data retention, information on sharing data, or making data available for future research.

### Plans for collection, laboratory evaluation, and storage of biological specimens for genetic or molecular analysis in this trial/future use {33}

Not applicable, as no biological specimens will be collected in this trial.

## Statistical methods

### Statistical methods for primary and secondary outcomes {20a}

All analyses will be performed blind by an independent researcher on the intention-to-treat (ITT) sample. We define the ITT sample as including all randomized participants (i.e., after the baseline assessment), irrespective of treatment compliance, withdrawal, or other protocol deviations. As per the CONSORT statement, baseline data will be presented by presenting mean and standard deviations for continuous variables (or median and a centile range when asymmetrically distributed) and by using numbers and proportions for categorical variables. Quantitative outcome measures will be analyzed using linear mixed modeling, using all five assessment time points of the primary outcome measure. A similar (but generalized) approach will be applied to estimate the impact on secondary outcomes. Our data, which is expected to contain missing values due to dropout, can be analyzed under the relatively relaxed assumption of missingness at random using linear mixed modeling [60, 61]. Mixed modeling will be used to evaluate the impact of condition (ABFT vs TAU) on SIQ-JR scores over all five measurements with participant ID and site as random effect. The fixed part of the model will have SIQ-JR as a function of time (discrete) and the interaction of condition by time [62]. Following the estimation of the mixed model, predictive marginal means will be computed and graphed in a margins plot to visualize the impact of condition on outcome over time. All analyses will be repeated for the secondary outcomes, adjusted where necessary to take into account characteristics (e.g., binary distribution) of these outcome measures.

The stratification variable (previous suicide attempts) will be examined as covariates in the analyses. Nonparametric Wilcoxon rank sum tests will be used to compare number of sessions attended. We will examine the effect of the level of family conflict on the effect of ABFT in an exploratory analysis (interaction/confounding effect).

#### CEA and CUA: general considerations

Both the cost-effectiveness analysis (CEA) and the cost-utility analysis (CUA) will be conducted in agreement with the latest Dutch guideline on economic evaluations [63] and the Belgian guideline for economic evaluations [64], hence from the societal and health care payer perspectives and in agreement with the ITT principle. Two base-case analyses will be presented, one according to each country-specific guideline. The CEA and CUA are conducted alongside the randomized trial and uses follow-up measurements post-intervention and at 3, 6, and 12 months after intervention.

#### CEA and CUA: cost calculations

Four types of costs will be included: (1) intervention costs (i.e., ABFT sessions), (2) costs stemming from health care utilization, (3) patients’ and their family’s costs for travel and informal care, and (4) costs stemming from productivity losses due to absenteeism and lesser efficiency while at work (i.e., presenteeism), both in paid work and volunteer jobs. For the Belgium perspective, we will only include health care costs paid out of the health care budget, by the federal government, the communities, and the patients, in line with the Belgian guideline. Data on resource use (health care uptake, informal care, travel distances to health services, and productivity losses) will be collected with the TIC-P [65]. The TIC-P is the most widely used health service utilization interview for economic evaluations in the Netherlands and can also be used in Belgium (note: in Belgium a slightly adapted version was used to better align with the Belgium healthcare system). Total costs will be estimated using a bottom-up (or micro-costing) approach, where units of health service are multiplied by their appropriate unit cost price and summed to provide an overall total cost estimate [66]. For the Dutch perspective, we shall make use of the standard unit cost prices as reported in the latest Dutch guideline for health economic evaluation [63, 67]. For the Belgian perspective, unit prices will be taken from sources referred to in the Belgian guidelines for economic evaluations (e.g., Rijksinstituut voor ziekte- en invaliditeitsverzekering; NomenSoft). Costs of medication (and dispensing costs) will be calculated using prices based on daily defined dosage (DDD) taken from www.farmacotherapeutischkompas.nl, www.medicijnkosten.nl, or https://www.riziv.fgov.be/, while accounting for the pharmacist’s claw back. Productivity losses will be based on the friction cost method as per the Dutch guideline and not included in the Belgium perspective. All costs will be expressed in 2026 euros. If necessary, existing cost prices will be updated to 2026 using the relevant national consumer price indices.

#### CEA and CUA: outcomes

For the CEA, the central clinical end-term will be a dichotomized version of the SIQ-JR, in which patients who score ≥ 31 will be categorized to be at potentially serious risk for suicide. For the CUA, the Dutch and Belgium tariffs (utility weights) of the EQ-5D-5L will be used for computing QALYs (cf. www.euroqol.org). Utility values will be calculated for these health states, using preferences elicited from the Dutch and Belgian population [54, 55].

Hence, results will be expressed as costs per proportion reduced case of potentially serious risk for suicide to treatment (in the CEA) and costs per QALY gained (in the CUA).

#### CEA and CUA: analysis

The comparability of groups at baseline will be assessed for both costs and outcomes. When necessary, methods will be applied to control for baseline differences [68, 69]. Missing cost and outcome data will be imputed using single imputation based on predictive mean matching nested in non-parametric bootstraps [70] for the ITT analysis. Since the trial’s follow-up measurements will not exceed 1 year, discounting will not be performed. Cumulative costs and QALYs over the study’s follow-up period will be computed with the area under the curve method to obtain the remission rate and cumulative QALY health gains as accrued over the measurements up to last follow-up at 12 months. The incremental cost-effectiveness ratio (ICER) will be computed to obtain the incremental costs per reduced case of potentially serious risk for suicide and the incremental costs per QALY gained. Stochastic uncertainty will be handled using 5000 non-parametric bootstraps and by plotting the simulated ICERs on the ICER-plane. For decision-making purposes, the cost-effectiveness acceptability curve will be plotted for various willingness-to-pay (WTP) ceilings for making judgments on whether adding ABFT offers good value for money relative to TAU. One-way sensitivity analyses directed at uncertainty in the main cost drivers and outcomes will be performed to assess the robustness of our findings (e.g., under different imputation strategies or using winsorization of high cost outliers). Both the analysis and reporting of the research findings will conform to the (extended) CONSORT and CHEERS statements [71–74].

### Interim analyses {21b}

No planned interim analyses will be performed. A stopping rule cannot be stated, but at any stage the safety board of the sponsor may request reconsideration of the trial. In case the study is ended prematurely, the investigator will notify the accredited MREC, including the reasons for the premature termination. There are no pre-defined criteria for ending the study.

### Methods for additional analyses (e.g., subgroup analyses) {20b}

There are no planned subgroup analyses for the primary analyses of interest.

### Methods in analysis to handle protocol non-adherence and any statistical methods to handle missing data {20c}

The study data is expected to contain missing values due to dropout and will therefore be analyzed under the relatively relaxed assumption of missingness at random using linear mixed modeling [60, 61]. In order to examine the impact of auxiliary variables (i.e., variables that are related to the missing data mechanism but not used in the model to answer the research question) on the results, a sensitivity analysis will be conducted in which missing data will be imputed using multiple imputation in which the number of imputations will be chosen equal to the percentage of missing data [75].

### Plans to give access to the full protocol, participant-level data, and statistical code {31c}

Pseudonymized data will be shared with the participating multicenter sites upon reasonable request and after discussion on the intentions for which the data will be used. There are otherwise no plans of granting public access to the full protocol, participant-level dataset, or statistical code.

## Oversight and monitoring

### Composition of the coordinating center and trial steering committee {5d}

NvdS, NB, LG, CB, and GB are responsible for conducting and coordinating the study, which includes any protocol revisions, the electronic case report forms, the reporting of serious adverse events, and the publication of study reports. NvdS, NB, and LG maintain weekly communication with the study sites and support both the local staff and the patients by answering questions and performing assessments. In addition, trained master-level psychology students will contribute by conducting interviews and supporting the team with administrative tasks.

Several stakeholders participate in the Trial Steering Committee together with the research team and the funder BeNeFIT. These are patient organizations Stichting Zelfbeschadiging, Depressievereniging, Ups & Downs, and suicide prevention organizations 113online and VLESP. The Trial Steering Committee convenes yearly to discuss the progress of the study.

### Composition of the data monitoring committee, its role and reporting structure {21a}

As there are no known harms associated with either the intervention or the control treatment, no other formal Data Safety Monitoring Board has been appointed.

### Adverse event reporting and harms {22}

There are no expected harms to come from participating in the study or from either of the treatment conditions. All adverse events (AE) reported spontaneously by the participants or observed by the investigator or site staff will be recorded. AEs are defined as any undesirable experience occurring to a subject during the study, whether or not considered related to the experimental intervention. We will also monitor and register serious adverse events (SAEs), especially fatal suicides, suicide attempts, and any medical occurrences (accidents, other causes of death, hospitalizations) that can mask suicide attempts. This will be monitored by the treating therapists and research coordinator at the study site, who will inform the sponsor when an SAE occurs. All AEs will be followed until they have abated, or until a stable situation has been reached. All SAEs will be reported to the accredited MREC that approved the protocol. AEs and SAEs are recorded using standardized forms that inquire about the event that has occurred, the data of the event, the outcome (i.e., ongoing at trial termination, recovered, resolved with sequelae, stabilized, or death), whether the event is related or not related to participation in the trial, whether the event is expected/anticipated or not, and whether any action was taken as a result of the event (i.e., none, temporary stop, permanent stop). Trial publications will report on all collected SAEs per treatment condition.

### Frequency and plans for auditing trial conduct {23}

All study procedures will be monitored by a monitor from the Clinical Monitoring Center (CMC) of Ziekenhuis Oost-Limburg (ZOL). The monitor is independent from the sponsor, the funder, and the participating study sites. The monitor will perform on-site or remote visits following a monitoring plan agreed upon with the sponsor, before the start of the inclusion period, during the inclusion period, and when all follow-up measurements at a specific site have taken place. The monitor will review details pertaining to data management, data administration, informed consent documents, inclusion and exclusion criteria, study procedures, and safety reporting.

### Plans for communicating important protocol amendments to relevant parties (e.g., trial participants, ethical committees) {25}

Any modification as a result of a protocol amendment approved by the MREC will be communicated to the local principal investigators of the study site who will inform their respective teams. If a modification affects participants already in the trial, they will be informed about the change and ask to give informed consent for remaining the trial. Important protocol modifications will also be outlined in any published reports at the end of the study.

## Dissemination plans {31a}

The results of the study will be shared with the participating mental health centers and in the relevant professional and scientific associations. The results will be presented on seminars and published in peer-reviewed journals. The results will not contain personal information.

## Discussion

ABFT has been disseminated in mental health care settings in various Western countries, to treat adolescents with suicidality, and has been increasingly used in emerging adults too [24, 25, 76]. ABFT is a family therapy intervention, targeting the attachment relationship with the caregivers, so that they can become a source of support and comfort in times of high distress and help break the isolation of the suicidal young adult.

Targeting attachment in suicidal youth is important. Because developing autonomy is a primary development task for emerging adults, and because they function increasingly independently of their parents, focusing therapy on repairing attachment bonds might seem an irrelevant and even an inappropriate intervention strategy in this age group. However, research points to the opposite. Autonomy development requires a secure attachment foundation (e.g., [77]). When trust is ruptured, youth fail to seek support which jeopardizes their ability to freely explore their environment, as this puts them at risk to be exposed to distress, they cannot manage. This impairs their autonomy development [78]. Emerging adults with severe mental health problems are often impaired in their attachment development, which makes it harder for them to develop autonomy and other competencies in this developmental phase [20]. These emerging adults struggle with their inability to develop the competencies that are considered normally acquired at this age. Moreover, due to the ruptures in their attachment bonds, they are not able to seek support for these struggles, eventually increasing their suicide risk.

However, despite some promising results of several RCTs, and the positive outcomes of pilot and feasibility studies on ABFT showing high satisfaction and symptom decrease, the evidence for the effectiveness of ABFT as a treatment for suicidality remains inconclusive (Schulte-Frankenfeld PM, Breedvelt JF, Brouwer ME, van der Spek N, Bosmans G, Bockting CL: Effectiveness of attachment-based family therapy for suicidal adolescents and young adults: a systematic review and meta-analysis, forthcoming). Further systematic research into the (cost-)effectiveness of ABFT for emerging adults is needed for at least three reasons. First, the previous studies are all efficacy studies, conducted in a research setting. Therefore, a pragmatic RCT is needed that examines the effectiveness of ABFT in a setting in which this care is actually delivered in daily practice. This level of evidence is also crucial and needed for decision-making regarding clinical guidelines for suicidality and depression. Second, ABFT was developed for adolescents in the North American culture and might not fully be transferable to emerging adults in the Netherlands and Belgium. Our pilot studies indicated the acceptability and feasibility of ABFT in the Netherlands and Belgium [20, 25, 27] and also for the young adult population. Nevertheless, an RCT is necessary to evaluate whether we indeed can yield similar effects in our culture. Third, worldwide the cost-effectiveness of ABFT, and to our knowledge any kind of family therapy, for suicidal patients has not yet been examined. This is important for dissemination policies. In addition, secondary analyses on mediators and predictors of effects can provide important knowledge on what types of treatments for suicidality work for whom. Regarding the mixed results found in previous studies, this study is crucial to examine the effectiveness and cost-effectiveness of ABFT, a promising family therapy intervention for suicidal emerging adults.

## Trial status

First approved protocol version in the Netherlands: Version 3, date May 8, 2023.

Latest approved protocol version in the Netherlands: Version 10, date June 10, 2024.

Start of recruitment: October 5, 2023.

Current status: Recruitment ongoing.

Approximate date of recruitment completion: May 2025.

## Supplementary Information

Supplementary Material 1.

## Data Availability

Researchers involved in the RCT from the study sponsor team (Amsterdam UMC) and the affiliated institutions (i.e., KU Leuven, ZOL, Trimbos Institute) will have access to the final trial dataset (pseudonymized data). The funding agency and the participating study sites can also request access to the pseudonymized data, upon reasonable request. The final trial dataset will not be publicly shared.

## Abbreviations

ABFT: Attachment-based family therapy
AE: Adverse event
CBT: Cognitive behavioral therapy
CDI-2: Children’s Depression Inventory
CEA: Cost-effectiveness analysis
CHEERS: Consolidated Health Economic Evaluation Reporting Standards
CMC: Clinical Monitoring Center
CONSORT: Consolidated Standards of Reporting Trials
CTQ: Childhood Trauma Questionnaire
CUA: Cost-utility analysis
DBT: Dialectical behavioral therapy
DDD: Daily defined dosage
DPIA: Data Protection Impact Assessment
E-SF: Entrapment Short-Form Scale
EAS: Emotional Autonomy Scale
(e)CRF: (Electronic) case report form
EDC: Electronic data capture
EQ-5D-5L: EuroQol-5 Dimensions-5 Levels
FAIR: Findability, accessibility, interoperability, and reusability
ICER: Incremental cost-effectiveness ratio
ITT: Intention-to-treat
MREC: Medical Research Ethics Committee
NSSI: Non-Suicidal Self-Injury Scale
PHQ-9: Patient Health Questionnaire
PSI: Psychological Separation Inventory
RCT: Randomized controlled trial
QALY: Quality-adjusted life year
SAE: Serious adverse event
SBS: Secure base script
SCID-5-S: Structured Clinical Interview for DSM-5
SIQ-JR: Suicidal Ideation Questionnaire Junior
SRFF: Self Report Measures of Family Functioning
TAU: Treatment as usual
TBRS-3: Therapist Behavior Rating Scale-3
TIC-P: Treatment Inventory of Costs in Patients
UMC: University Medical Center
VLESP: Vlaams Expertisecentrum Suïcidepreventie
WHO: World Health Organization
WHODAS 2.0: World Health Organization Disability Assessment 2.0
WTP: Willingness-to-pay
ZOL: Ziekenhuis Oost-Limburg
