# Supplementary material for: The effectiveness and cost-effectiveness of attachment-based family therapy for young adults with high suicidal ideation: protocol of a randomized controlled trial
Source: Trials. 2024 Oct 16;25:686. doi: 10.1186/s13063-024-08499-7 (PMC11484370; doi:10.1186/s13063-024-08499-7)
Supplement: Supplementary file 1 — Supplementary Material 1. [file 13063_2024_8499_MOESM1_ESM.docx]

**Appendix A: Overview Study Sites**

| **België** | **Nederland** |
| --- | --- |
| 1. PraxisP KU Leuven | 1. AMC, psychiatry department, outpatient clinic for young adults with affective disorders, Amsterdam |
| 1. Studentengezondheidscentrum KU Leuven (STUVO Psychologen en Psychiaters) | 1. Levvel, Amsterdam |
| 1. UGent Centrum Kind en Adolescent | 1. GGZ Oost Brabant, Depressie expertisecentrum jeugd, Oss |
| 1. Praktijk Annemie Uyttersprot | 1. GgzE, Eindhoven |
| 1. UPC KU Leuven, campus Kortenberg | 1. Arkin Jeugd en Gezin, Amsterdam |
| 1. Praktijk ConnectUs Tongeren | 1. GGZ Centraal, Amersfoort |
|  | 1. Kenter Jeugdhulp, Santpoort-Noord |

**Appendix B: Study Materials – Informed Consent Letters**

**JONGVOLWASSENE**

**OUDER/VERZORGER**

Informed Consent Letter Young Adults – Belgium

**REPAIR project: Treating suicidality in young adults: a study on Attachment Based Family Therapy**

Sponsor of the study: Amsterdam UMC, Meibergdreef 9, 1105 AZ Amsterdam

Medical Ethics Committee: *Identification of the Ethics Committee that issued the single opinion on the trial and the local Ethics Committee that took part in the approval process.*

Local investigators: *Name, affiliation and contact details*

# I Information vital to your decision to take part

**Introduction**

You receive this letter because you suffer from suicidal thoughts for which you receive or seek treatment. You are invited to participate in a clinical trial evaluating a psychotherapy for the treatment of suicidality.

The sponsor and investigator hope that this psychotherapy will offer benefits for the treatment of people who suffer from suicidality. However, there is no guarantee that your participation in this study will benefit you.

Before you agree to take part in this study, we invite you to take note of its implications in terms of organisation, possible risks and benefits, to allow you to make a decision with full awareness of the implications. This is known as giving “informed consent”.

Please read these few pages of information carefully and ask any questions you want to the investigator or his/her representative. There are 3 parts to this document: the information essential to your decision, your written consent and supplementary information (appendices) detailing certain aspects of the basic information.

**If you take part in this clinical study, you should be aware that:**

1. This clinical study is being conducted after having been reviewed by one or more ethics committees.
2. Your participation is voluntary and must remain free from any coercion. It requires the signature of a document expressing your consent. Even after having signed this document, you can stop taking part by informing the investigator. Your decision not to take part or to stop taking part in the study will have no impact on the quality of your care or on your relationship with the investigator.
3. The data collected on this occasion are confidential and the protection of your identity will be guaranteed during publication of the results.
4. During treatment we will record therapy sessions with your and your parent(s')/caregiver(s') consent if you are drawn into the study condition in which family therapy (Attachment Based Family Therapy, explanation see p. 2) is given. The reason for this is that there is a test to be done that verifies whether the family therapy was given as it was intended and thus was carried out correctly by the practitioner/therapist. So this test is done to prove that you and your parent(s)/caregiver(s) actually received Attachment Based Family Therapy. We only do recording of sessions with written consent.
5. You will not be charged for specific treatments, visits, examinations in the context of this study. You and/or your parent(s)/caregiver(s) can only be charged for costs related to usual medical/psychological performance in your clinical situation.
6. Insurance (see appendix D) has been taken out in case you should suffer any damage in connection with your participation in this clinical study.
7. You may contact the investigator or a member of his/her team at any time should you need any additional information.

Further information about your “Rights as a participant in a clinical study” can be found in appendix B.

**Objectives and description of the study protocol**

This research was set up by the Catholic University of Leuven and the AMC (Amsterdam Medical Centre). Below, we always call AMC the ‘sponsor’. We invite you to participate in a clinical intervention study on psychotherapy treatment (Attachment Based Family Therapy; ABFT) in approximately 138 participants, of whom about 69 in Belgium.

Attachment Based Family Therapy is an intervention developed in America with the goal of reducing suicidal thoughts and behaviors by identifying and repairing fractures in the parent-child relationship, making it easier for young people to seek help during stress and thus less likely to desire suicide. At ABFT, the young adult and parent(s)/caregiver(s) receive approximately 16 treatment sessions (this may be slightly less, or slightly more), spread over 16 weeks.

Suicidal thoughts among young adults are common. Standard treatment for these complaints is the initiation of psychological treatment, possibly in combination with medication. There is now evidence that Attachment Based Family Therapy is successful in reducing suicidal thoughts in young adults.

The ABFT will be compared with other treatments offered in the Netherlands and Belgium. These can be different treatments. Attachment Based Family Therapy is seen as one of the standard treatments for suicide cases in the Netherlands and Belgium. However, it has never been investigated in the Netherlands or Belgium whether ABFT is more or as successful as the other treatments. This is what we are going to do in this REPAIR project. REPAIR stands for REbuilding Parent young Adult Interpersonal Relationships. Researchers, these can be researchers, psychologists and psychiatrists, conduct the research in different institutions in Belgium and the Netherlands.

*Are you suitable to participate?*

We first want to know if you are suitable to participate. That is why the researcher first does an inter-view of about 15 minutes, after you have signed the information and consent form.

In order to participate, it is important to know what psychological symptoms you are experiencing and to what extent. For this purpose, a brief preliminary examination will take place, face to face, by telephone or via video call. You can indicate your preference. If after this preliminary examination it appears that you can participate in the study and you and your parent(s)/caregiver(s) indicate in this information and consent letter that you want to participate, you and your parent(s)/caregiver(s) will receive a number of questionnaires. The questionnaires will be about your symptoms/symptoms, attachment, family functioning or the relationship with the therapist in the study (during the period of therapy). The time it will take to complete the questionnaires can be found in the table p. 4.

To participate in the study, you must:

a) be between 18 and 25 years old;

b) have a score of 31 or above on the SIQ-JR (This is the Suicidal Ideation Questionnaire, a questionnaire to estimate the degree of suicidality in a given person; when the score is 31 or more, we speak of suicidality) ;

c) have at least one parent or caregiver participating in the assessment and treatment. This can be a biological parent, stepparent, grandparent, other relative or foster parent.

Exclusion criteria are (criteria that result in your inability to participate in the study):

a) other primary disorders: severe alcohol or cannabis use disorder, all other substances: moderate or severe substance use disorder, severe conduct disorder, evidence of psychotic features or prior psychosis;

b) severe cognitive impairment (e.g. mental retardation, severe developmental disorders) as evidenced by educational records, parental reports and/or clinical impressions;

c) other circumstances that may affect participation (e.g. serious medical condition, relocation).

**Course of the study**

*How long does the investigation /study take?*

Your participation in the study will take approximately 17 months.

*Step 1: Are you suitable to participate?*

We first want to know if you are suitable to participate. That is why the researcher first does an inter-view of about 15 minutes, after you have signed the information and consent form.

In order to participate, it is important to know what psychological symptoms you are experiencing and to what extent. For this purpose, a brief preliminary examination will take place, face to face, by telephone or via video call. You can indicate your preference. If after this preliminary examination it appears that you can participate in the study and you and your parent(s)/caregiver(s) indicate in this information and consent letter that you want to participate, you and your parent(s)/caregiver(s) will receive a number of questionnaires.

*Step 2: the treatment*

You will receive treatment for your suicidal thoughts and additional symptoms as part of the study. This treatment will be followed up by the research team for 5 months. If treatment can be completed after these 5 months, it will be completed. If the practitioner/therapist deems it necessary that further treatment is needed, either the treatment will be continued or a suitable next treatment will be sought, so that you do not simply fall without treatment.

For this research we make 2 groups:

• Group 1. The people in this group receive ABFT in addition to standard care. ABFT involves parents/caregivers. If you are assigned to group 1, your parent(s)/caregiver(s) will also participate in the therapy sessions. Furthermore, you will complete questionnaires as part of the study.

• Group 2. The people in this group receive standard care. Standard care is the care currently indicated for the treatment of suicidality in young adults. Usually this is some form of therapy, such as Cognitive Behavioral Therapy, combined with medication. Those in this group will not receive ABFT, so parents/caregivers will not be involved in therapy sessions. The parents/caregivers do participate in the study, which means that they will answer questionnaires.

A draw determines which treatment you receive.

If you are receiving ABFT treatment, we will ask your permission and that of your parent(s)/caregiver(s) to videotape the therapy sessions. These video recordings are used by researchers to determine whether the ABFT treatment was given as intended. In this video, we look primarily at the practitioner/therapist giving the therapy/treatment, to verify that this therapist actually applied/gave the ABFT as it was intended. You and your parent(s)/caregiver(s) will be given the choice of whether or not to be filmed recognizably The video recordings will be securely stored at your institution's location and on a secure drive (Research Drive of the AMC) of the client. Only the researchers will have access to these video recordings. After the researchers have reviewed the video recordings, they are promptly deleted/destroyed. This ensures the protection of the identity of you and your parent(s)/caregiver(s). Under no circumstances will the video footage be shown to students for educational purposes and there will be no marketing or other commercial purposes. If you agree, please check this on the consent form. You and your parent(s)/caregiver(s) have the right to indicate at any time that you do not wish to participate in this study or no longer wish to be filmed during therapy. This will then not affect further participation in the study.

*Step 3: examinations and measurements*

We will send you a questionnaire 5 times and your parent(s) or caregiver(s) 4 times. The questions are about your complaints and suicidal thoughts, attachment, family functioning or the relationship with the therapist in the study. It will take you about 30 minutes to complete this questionnaire. The questionnaire for your parent(s)/carer(s) takes about 10 minutes to be completed.

The researcher will also call you a total of 4 times, or he/she will make an appointment with you at the location of the institution where you are receiving treatment. You will then receive questions about your current suicidality complaints. This conversation lasts about 30 minutes.

If you and your parent(s)/caregiver(s) decide to participate in the study and you meet all the conditions for participation, you and your parent(s)/caregiver(s) will go through the following tests and studies:

Assessments for young adult

| **Appointment** | **Time** | **Activities** | **Duration** | **Location** |
| --- | --- | --- | --- | --- |
| Eligibility screening | After intake/after signing  Consent  form -  Informed Consent | Interview | 15 minutes | At home/ online, or at your treatment facility |
| Measurement 1 | After eligibility screening | Interview and questionnaires | 60 minutes | At home/ online, or at your treatment facility |
| Intervention | During first session, every two months, and last session | Questionnaire | 3 minutes per session | At home/ online, or at your treatment facility |
| Measurement 2 | After end of treatment | Interview and questionnaires | 60 minutes | At home/ online, or at your treatment facility |
| Measurement 3 | 3 months after treatment | Interview and questionnaires | 60 minutes | At home/ online, or at your treatment facility |
| Measurement 4 | 6 months after treatment | Questionnaires | 10 minutes | Online |
| Measurement 5 | 12 months after treatment | Interview and questionnaires | 60 minutes | At home/ online, or at your treatment facility |

Assessment for the parent(s)/caregiver(s)

| **Appointment** | **Time** | **Activities** | **Duration** | **Location** |
| --- | --- | --- | --- | --- |
| Measurement 1 | After eligibility screening of your child and informed consent | Questionnaires | 10 minutes | Online |
| Measurement 2 | After end of treatment | Questionnaires | 10 minutes | Online |
| Intervention | During first session, every two months, and last session | Questionnaire | 3 minutes per session | Online |
| Measurement 3 | 3 months after treatment | Questionnaires | 10 minutes | Online |
| Measurement 4 | 12 months after treatment | Questionnaires | 10 minutes | Online |

**Risks and inconveniences**

**What side effects, adverse effects or discomforts can you suffer from?**

There are no major risks associated with ABFT and standard care. However, it is possible, however, that completing the questionnaires may cause some discomfort to you and/or your parent(s)/caregiver(s) and thus may be emotionally taxing. All study participants will be given a handout if they are included, which lists help sites such as the helpline/suicide line (1813 in Belgium). Participants can also contact their practitioner/therapist in the study at any time.

Another disadvantage of the study is that it takes up time from you and your parent(s)/caregiver(s). The appointments that take place as part of this study will be scheduled according to your preference and that of your parent(s)/caregiver(s) as much as possible.

Possible side effects of your standard treatment:

- Psychological treatment: there are no risks associated with psychological treatment. However, the therapy may be emotionally taxing for you.
- Medication: see package insert of the medication you are taking for specification of side effects.

**What are the advantages and disadvantages of participating in the study?**

Participating in the research can have advantages and disadvantages. Below we list them. Think about this carefully, and talk about it with others.

The treatment ABFT can further reduce your suicidal thoughts and additional complaints, but that is not certain. At any time during this examination, these complaints may come back or worsen. If symptoms worsen or a crisis situation occurs, practitioners/therapists and investigators always consult the crisis protocol, which describes procedures on what to do in a crisis situation. In this way, appropriate care will always be offered.

Participating in the study can have these disadvantages:

- You may experience the side effects or adverse effects described above.
- Participating in the study will cost you extra time.
- You must adhere to the agreements associated with the investigation.

*Do you or your parent(s)/caregiver(s) not want to participate?*

It is your choice whether or not you wish to participate in this study. Do you not wish to participate? Then you will receive the standard treatment for your suicidal thoughts. Your therapist/practitioner can tell you more about the available options for treatment and about their pros and cons.

**When does the study end?**

The investigator will let you know if there is any new information about the study that is important to you. The investigator will then ask you if you want to continue to take part.

In the following situations, the study will stop for you:

- All check-ups according to the schedule are finished.
- The end of the study has been reached.
- You want to stop participating in the study. You can stop at any time. Report this to the investigator immediately. You do not have to explain why you want to stop. You will continue to receive treatment for your suicidal thoughts and co-morbid symptoms. The investigator will still invite you for a follow-up check.
- The investigator thinks it is better for you to stop. The investigator will still invite you for a follow-up check.
- One of the following authorities decides that the study should stop:
  - AMC (prof. Bockting), or
  - the government, or
  - the Medical Ethics Review Committee assessing the study

*What happens if you stop participating in the study?*

If you withdraw your consent to participate in the study, the coded data already collected before your withdrawal will be retained. This will ensure the validity of the study. No new data will be transmitted to the sponsor.

The entire study ends when all the participants have finished their treatments and check-ups.

**Treatment after discontinuation of the study**

In all situations where participation in the study is stopped, but also when the study is completed as planned, the researcher/therapist will examine your health and prescribe you the best treatment available. So you will still receive your treatment for suicidal thoughts and additional complaints. The researcher/therapist can arrange one or more checks for your safety.

**What happens after the study?**

*Will you receive the results of the study?*

After processing all the data, about a year after your participation, the researcher will let you know what the most important results of the study are.

**If you participate in this study, we ask you the following:**

We would like the research to go well. That is why we make the following agreements with you:

- You and your parent/carer follow the treatment as agreed with your practitioner/therapist.
- You come to every appointment.
- You contact the researcher in these situations:
  - You will be hospitalized or treated.
  - You suddenly have problems with your health.
  - You no longer want to participate in the study.
  - Your phone number, address or email address will change.

**You should also know that:**

- in order to participate in this clinical trial and for your own safety, you must agree that the investigator informs the various physicians, therapists and other specialists involved in your treatment about your participation in this clinical trial. In case of increasing suicidal thoughts or other crisis situations, we can then contact your general practitioner and / or treating specialist, for example about the status of your current treatment.

**What will happen to your data?**

*What data do we store?*

We store the following data:

- your name

- your gender

- your address

- your date of birth

- information from your parent(s)/caregiver(s)

- information about your health

- (medical) information that we collect during the study

*Why do we collect, use and store your data?*

We collect, use, and store your data to answer the questions of this study and to be able to publish the results.

*How do we protect your privacy?*

To protect your privacy, your data will be given a code. We only use this code for your personal data. We keep the key to the code in a safe place at the facility/institution where you are being treated at, , so only at the participating site.. When we process your data, we always use only that code. Your name or personal data will never appear in any report or publication about this research. The coded data will be securely shared with the statistical experts, who are associated with our study, to analyze the (cost)-effectiveness of the treatment.

*Who can see your data?*

In order to control the quality of the study, it is possible that your non-coded personal data or information from your medical record relevant to this study may be inspected by people other than the study staff. This inspection is done under the supervision of the researcher and these people are bound by professional secrecy or through a confidentiality agreement. They may be:

- personnel designated by the client (MONITORS and AUDITORS) and people or organizations providing services to or working with the client. However, they will never disclose your name and con-tact information to the client.

- inspectors from competent health authorities around the world
- an independent audit group
- persons appointed by the Ethics Committee

*How long do we keep your data?*

After the study ends, your coded data will be kept for at least 25 years (Ref. 1) to ensure the validity of the study. This will be the case even if you stop participating in the study early. The data may still be viewable in the context of an audit or inspection by the government or other authorities in connection with the treatment examined. As soon as this is no longer necessary, we will destroy your data.

By giving permission to participate in this study, you also agree that your coded data from this study can be used by the grant providers (BeNeFIT call funders, being KCE in Belgium and ZonMw in the Netherlands) or similar public health research institutes in Europe for further analyses. For example, to determine whether one of the treatments examined offers an additional value.

Under no circumstances will the researchers carrying out the additional analyses see your identity and all researchers are bound by professional secrecy.

*Can we use your data for other research?*

At the end of this examination, your data may also be important for **other scientific research** on suicidal thoughts and treatments. This study remains within the context of the current clinical trial and thus aims to better understand your symptoms, their treatment and response to this treatment. For this purpose, your data will be stored with at the sponsor site for 25 years.

*What happens with accidental finds?*

A result found by chance during the study and on top of the objectives is called an accidental find. If this result may be important to your health or that of your blood relatives, the sponsor will inform the investigator. With your consent, the researcher will inform you and your practitioner/therapist/treating physician of your results and the possible consequences. If necessary, the researcher and/or practitioner/therapist/treating physician will advise you on what to do.

You agree or disagree to be informed by checking the appropriate box on page 10.

*What happens to your data if you no longer wish to participate in the study?*

You can withdraw your participation in the study at any time. But please note: do you decide to stop the study, and have researchers already collected data for a study? Then they may still use this data.

*Would you like to know more about your privacy?*

- Would you like to know more about your rights when processing personal data? Then take a look at [www.gegevensbeschermingsautoriteit.be](http://www.gegevensbeschermingsautoriteit.be)
- Do you have questions about your rights? Or do you have a complaint about the processing of your personal data? Please contact the person responsible for processing your personal data. For your research, that is:
  - KU Leuven. See Appendix A for contact details, and website.
- If you have any complaints about the processing of your personal data, we recommend that you first discuss them with the research team. You can also report this to the Belgian supervisory authority which monitors compliance with the basic principles of the protection of personal data (see Appendix A).

*Where can you find more information about the study?*

You can find more information about the study on the following website: <https://repairstudy.com>

**Will you receive compensation if you participate in the study?**

The tests and treatment for the study will not cost you anything. You will receive a voucher of €15,- per assessment. The four assessments for which you will receive a voucher are the initial assessments, immediately after the treatment, and the ones 3 and 12 months after the treatment. You will receive €5.- for the online questionnaire 6 months after treatment. In total, you can earn €65,- by participating in all 5 assessments. This does not apply to parent(s)/caregiver(s). Parent(s)/caregiver(s) receive €8 per assessment, which amounts to a maximum of €32,- in total. If you or your parent(s)/caregiver(s) stop before the study is finished, the compensation you receive will be less.

### Are you insured during the study?

Any participation in a clinical study involves a risk, however small it is. Even if there is no fault, the sponsor accepts responsibility for damage caused to the participant (or in the event of death, his/her dependants) and directly or indirectly linked to his/her participation in the study. The sponsor has taken out insurance for this responsibility^[[1]](#footnote-1)^.

You are therefore asked to report any new health problem to the investigator before consulting another therapist, taking any other medication or receiving any other therapeutic treatment. If, for any reason, you consult another therapist during this clinical study, you must inform him/her that you are taking part in a clinical study. This could be important in establishing a diagnosis and treating your complaints.

If the investigator believes that a link with the study is possible (the insurance does not cover the natural progression of your disease or the known side effects of your normal treatment), he/she will inform the study sponsor, which will initiate the declaration procedure to the insurance company. The latter will appoint an expert - if it considers it necessary - to assess whether there is a link between your new health problems and the study.

In the event of disagreement either with the investigator or with the expert appointed by the insurance company and also whenever you feel it is appropriate, you or - in case of death - your dependants may bring proceedings against the insurer directly in Belgium (name of insurance company, policy number, contact).

The law provides that the insurer may be summoned to appear either before the judge of the location where the event giving rise to the damage occurred, or before the judge of your domicile, or before the judge of the insurer’s registered offices.

**Contact**

If you need further information, but also if you have problems or concerns, you can contact the investigator (Surname, First name) or a member of his/her research team (Surname, First name) on the following telephone number (xx / xxx-xx-yy).

In case of emergency, you can contact XX on the following telephone number XX.

If you have any questions relating to your rights as a participant in a study, you can contact the ethics committee on this telephone number: +32 16 34 86 00 (weekdays from 10 a.m. to 11 a.m.).

**How do you give consent for the study?**

You can first carefully think about this study. Then you tell the investigator of the KU Leuven (independent of the health care provider) if you understand the information and if you want to take part or not. If you and your parent(s)/caregiver(s) want to take part, fill in the consent form that you can find with this information sheet. You, your parent(s)/caregiver(s), and the investigator will get a signed version of this consent form.

Thank you for your time.

**REPAIR project: Treating suicidality in young adults: a study on Attachment Based Family Therapy**

# II Informed consent

**Participant**

I declare that I have been informed of the nature of the study, its purpose, its duration, any risks and benefits and what is expected of me. I have taken note of the information document and the appendices to this document.

I have had sufficient time to think about it and discuss it with a person of my choice, such as my GP or a member of my family.

I have had the opportunity to ask any questions that came to mind and have obtained a satisfactory response to my questions.

I understand that my participation in this study is voluntary and that I am free to end my participation in this study without this affecting my relationship with the therapeutic team in charge of my health.

I understand that data about me will be collected throughout my participation in this study and that the investigator and the sponsor of the study will guarantee the confidentiality of these data in accordance with applicable European and Belgian legislation.

I agree to my personal data being processed as described in the section dealing with confidentiality guarantees (appendix B). I also consent to these data being transferred to and processed in countries other than Belgium.

I agree to my GP or other specialists in charge of my health being informed of my participation in this clinical study.

I give consent to request information from my GP and therapist about my suicidal thoughts and psychological symptoms.

I give consent to give my doctor, specialist or therapist information about accidental discoveries that may occur during the study that are important for my health.

I know that some people will be able to see all of my data to review the study. These people are mentioned in this information sheet. I give consent to let them see my data for this review.

I have received a copy of the information provided to the participant and the informed consent.

Please tick yes or no in the table below.

| I give permission for our therapy sessions to be videotaped (where I have the choice of being filmed recognizably or not on the video footage). | Yes ☐ | No  ☐ |
| --- | --- | --- |
| I give permission for the interview as part of the Secure Base Script Task (attachment interview) to be video or audio recorded. | Yes ☐ | No  ☐ |
| I give consent to ask me after this study if I want to participate in a follow-up study. | Yes ☐ | No  ☐ |
| I agree that in case of an accidental finding, the researcher will notify the practitioner/therapist/treating physician and myself of this and the possible consequences. | Yes ☐ | No  ☐ |
| I consent to my e-mail address being used in the electronic data system to send me the questionnaires and that my telephone number and address data are used in case this is necessary. | Yes ☐ | No  ☐ |

Surname, first name, date and signature of the volunteer.

**Investigator**

I, the undersigned, [surname, first name] investigator/clinical study assistant, confirm that I have verbally provided the necessary information about the study and have given the participant a copy of the information document.

I confirm that no pressure was applied to persuade the patient to agree to take part in the study and that I am willing to answer any additional questions if required.

I confirm that I operate in accordance with the ethical principles set out in the latest version of the “Helsinki Declaration”, the “Good Clinical Practices” and the Belgian Law of 7 May 2004 related to experiments on humans.

Surname, first name, date and signature

of the investigator’s representative

**REPAIR project: Treating suicidality in young adults: a study on Attachment Based Family Therapy**

# III Supplementary information

**Appendix A: contact information of Amsterdam UMC**

**Contact details of the participating site**

Principal Investigator at the site:

Coordinator at site:

Therapists at the site:

**Principal investigator:**

Professor Claudi Bockting

Meibergdreef 9

1105 AZ Amsterdam

Tel: +3120-8913600

E-mail: [c.l.bockting@amsterdamumc.nl](mailto:c.l.bockting@amsterdamumc.nl)

**Researchers**:

Dr. Nadia van der Spek

Meibergdreef 9

1105 AZ Amsterdam

Tel: +3120-8913600

E-mail: [n.vanderspek@amsterdamumc.nl](mailto:n.vanderspek@amsterdamumc.nl)

Professor Guy Bosmans

KU Leuven

Tiensestraat 102, 3000 Leuven, Belgium

Department of clinical psychology

Tel: +3216326187

E-mail: [guy.bosmans@kuleuven.be](mailto:guy.bosmans@kuleuven.be)

Luana Gavan

Meibergdreef 5

1105AZ Amsterdam

Tel: +3120-8913671

Email: [l.gavan@amsterdamumc.nl](mailto:l.gavan@amsterdamumc.nl)

Nele Bergers

KU Leuven

Tiensestraat 102, 3000 Leuven, Belgium

Department of clinical psychology

Tel: +3216326187

Email: [nele.bergers@kuleuven.be](mailto:nele.bergers@kuleuven.be)

**Complaints with regard to the processing of personal data**

The Belgian supervisory authority is called:

Gegevensbeschermingsautoriteit (GBA)

Drukpersstraat 35,

1000 Brussel

Tel. +32 2 274 48 00

e-mail: contact(at)apd-gba.be

Website: [www.gegevensbeschermingsautoriteit.be](http://www.gegevensbeschermingsautoriteit.be)

**Appendix B: Supplementary information on the protection and the rights of the participant in a clinical study**

### *Ethics Committee*

This study has been reviewed by an independent Ethics Committee, namely the Ethics Committee of [Name of EC], which has issued a favourable opinion [*after consulting with the Ethics Committees of each centre where this trial will be conducted*]. It is the task of the Ethics Committees to protect people who take part in a clinical trial. They make sure that your rights as a patient and as a participant in a clinical study are respected, that based on current knowledge, the balance^[[2]](#footnote-2)^ between risks and benefits remains favourable to the participants, that the study is scientifically relevant and ethical.
You should not under any circumstances take the favourable opinion of the Ethics Committee as an incentive to take part in this study.

### *Voluntary participation*

Before signing, do not hesitate to ask any questions you feel are appropriate. Take the time to discuss matters with a trusted person if you so wish.

Your participation in the study is voluntary and must remain free of any coercion: this means that you have the right not to take part in the study or to withdraw without giving a reason, even if you previously agreed to take part. Your decision will not affect your relationship with the investigator or the quality of your future therapeutic care.

However, it is advisable for your safety to inform the investigator if you have decided to stop taking part in the study.

If you agree to take part, you will sign the informed consent form. The investigator will also sign this form to confirm that he/she has provided you with the necessary information about the study. You will receive a copy of the form.

### *Costs associated with your participation*

### If you decide to participate in this study, this will not entail any additional costs for you, your parent(s)/caregiver(s) or your insurance company. The visits and procedures indicated in the description of the conduct of the investigation on pages x1 to x2/y as specific to the investigation are the responsibility of the sponsor. Only costs related to usual medical/psychological performance in your clinical situation can be charged to you. So you and/or your parent(s)/caregiver(s) only pay the costs that you would normally pay for a treatment.

### *Guarantee of confidentiality*

Your participation in the study means that you agree to the investigator collecting data about you and to the study sponsor using these data for research purposes and in connection with scientific and medical publications.

The processing of your personal data is necessary to achieve the scientific research purposes as set out herein. We would like to inform you that the necessity of the processing for the conduct of scientific research as a task of public interest constitutes the lawful basis on which we process your information in the context of the study in which you are participating.

Your data will be processed in accordance with the European General Data Protection Regulation (GDPR) and with the Belgian legislation on the protection of natural persons with regard to the processing of personal data. . Academisch Medisch Centrum, Amsterdam shall act as data controller for your data.

The legal basis for processing your data is the public interest. This means that the research will lead to an increase in knowledge and insight that will benefit society (directly or indirectly).

You are entitled to ask the investigator what data are being collected about you and what is their use in connection with the study. This data concerns your current clinical situation but also some of your background, the results of examinations carried out within the context of care of your health in accordance with the current standards and obviously the results of examinations required by the protocol. You have the right to inspect these data and correct them if they are incorrect^[[3]](#footnote-3)^.

The investigator has a duty of confidentiality vis-à-vis the data collected.

This means that he/she undertakes not only never to reveal your name in the context of a publication or conference but also that he/she will encode (your identity will be replaced by an ID code in the study) your data before sending them to the manager of the database of collected data (controller: AMC, location of the database: on the AMC's secure L or G drive ).

The therapists at the treating sites and investigator and his/her team will therefore be the only ones to be able to establish a link between the data transmitted throughout the study and your medical records^[[4]](#footnote-4)^.

The personal data transmitted will not contain any combination of elements that might allow you to be identified^[[5]](#footnote-5)^.

For the study data manager designated by the sponsor, the data transmitted will not allow you to be identified. The latter is responsible for collecting the data gathered by all investigators taking part in the study, processing them and protecting them in accordance with the requirements of the Belgian law on the protection of privacy.

To verify the quality of the study, it is possible that your medical records will be examined by persons subject to professional secrecy and designated by the ethics committee, the sponsor of the study or an independent audit body. In any event, this examination of your medical records may only take place under the responsibility of the investigator and under the supervision of one of the collaborators designated by him/her.

The (encoded) study data will be able to be sent to Belgian or other regulatory authorities, to the relevant ethics committees, to other doctors and/or to organisations working in collaboration with the sponsor.

They will also be able to be sent to other sites of the sponsor in Belgium and in other countries where the standards in terms of the protection of personal data may be different or less stringent? As explained above, the transmitted data are encoded.^[[6]](#footnote-6)^

Your consent to take part in this study therefore also implies your consent to the use of your encoded medical data for the purposes described in this information form and to their transmission to the aforementioned people and authorities.

The sponsor will use the data collected within the context of the study in which you are taking part, but would also like to be able to use them in connection with other research concerning the same psychological difficulties as yours. Any use of your data outside the context described in this document is only possible with the approval of the ethics committee.

If you withdraw your consent to take part in the study, to guarantee the validity of the research, the data encoded up to the point at which you withdraw will be retained. No new data may be sent to the sponsor.

If you have any questions relating to how your data are being processed, you may contact the investigator. The data protection officer in your hospital can be contacted as well: DPO - UZ Leuven, Herestraat 49, 3000 Leuven, e-mail [dpo@uzleuven.be](mailto:dpo@uzleuven.be).

Finally, if you have a complaint concerning the processing of your data, you can contact the Belgian supervisory authority who ensures that privacy is respected when personal data are processed.

The Belgian supervisory authority is called:

Data Protection Authority (DPA)

Drukpersstraat 35,

1000 Brussels

Tel. +32 2 274 48 00

e-mail: contact@apd-gba.be

Website: <https://www.dataprotectionauthority.be>

### *Insurance*

Any participation in a clinical study involves a risk, however small it is. Even if there is no fault, the sponsor accepts responsibility for damage caused to the participant (or in the event of death, his/her dependants) and directly or indirectly linked to his/her participation in the study. The sponsor has taken out insurance for this responsibility^[[7]](#footnote-7)^.

You are therefore asked to report any new health problem to the investigator before consulting another therapist, taking any other medication or receiving any other therapeutic treatment. If, for any reason, you consult another therapist during this clinical study, you must inform him/her that you are taking part in a clinical study. This could be important in establishing a diagnosis and treating your complaints.

If the investigator believes that a link with the study is possible (the insurance does not cover the natural progression of your disease or the known side effects of your normal treatment), he/she will inform the study sponsor, which will initiate the declaration procedure to the insurance company. The latter will appoint an expert - if it considers it necessary - to assess whether there is a link between your new health problems and the study.

In the event of disagreement either with the investigator or with the expert appointed by the insurance company and also whenever you feel it is appropriate, you or - in case of death - your dependants may bring proceedings against the insurer directly in Belgium (name of insurance company, policy number, contact). The law provides that the insurer may be summoned to appear either before the judge of the location where the event giving rise to the damage occurred, or before the judge of your domicile, or before the judge of the insurer’s registered offices.

Informed Consent Letter Parent(s)/caregiver(s) – Belgium

**REPAIR project: Treating suicidality in young adults: a study on Attachment Based Family Therapy**

Sponsor of the study: Amsterdam UMC, Meibergdreef 9, 1105 AZ Amsterdam

Medical Ethics Committee: *Identification of the Ethics Committee that issued the single opinion on the trial and the local Ethics Committee that took part in the approval process.*

Local investigators: *Name, affiliation and contact details*

# I Information vital to your decision to take part

**Introduction**

You receive this letter because your child suffers from suicidal thoughts for which you receive or seek treatment, or because your child has suicidal thoughts and is seeking or receiving treatment for them. You are invited to participate in a clinical trial evaluating a psychotherapy for the treatment of suicidality.

The sponsor and investigator hope that this psychotherapy will offer benefits for the treatment of people who suffer from suicidality. However, there is no guarantee that participation in this study will benefit your child.

Before you agree to take part in this study, we invite you to take note of its implications in terms of organisation, possible risks and benefits for you and your child, to allow you to make a decision with full awareness of the implications. This is known as giving “informed consent”.

Please read these few pages of information carefully and ask any questions you want to the investigator or his/her representative. There are 3 parts to this document: the information essential to your decision, your written consent and supplementary information (appendices) detailing certain aspects of the basic information.

**If you take part in this clinical study, you should be aware that:**

1. This clinical study is being conducted after having been reviewed by one or more ethics committees.
2. Your participation is voluntary and must remain free from any coercion. It requires the signature of a document expressing your consent. Even after having signed this document, you can stop taking part by informing the investigator. Your decision not to take part or to stop taking part in the study will have no impact on the quality of your care or on your relationship with the investigator.
3. The data collected on this occasion are confidential and and the protection of your identity will be guaranteed during publication of the results.
4. During treatment we will record therapy sessions with your and your child’s consent if you and your child are drawn into the study condition in which family therapy (Attachment Based Family Therapy, explanation see p. 2) is given. The reason for this is that there is a test to be done that verifies whether the family therapy was given as it was intended and thus was carried out correctly by the practitioner/therapist. So this test is done to prove that you and your child actually received Attachment Based Family Therapy. We only do recording of sessions with written consent.
5. You will not be charged for specific treatments, visits, examinations in the context of this study. You and/or your child can only be charged for costs related to usual medical/psychological performance in the clinical situation of your child.
6. Insurance (see appendix D) has been taken out in case you should suffer any damage in connection with your participation in this clinical study.
7. You may contact the investigator or a member of his/her team at any time should you need any additional information.

Further information about your “Rights as a participant in a clinical study” can be found in appendix D.

**Objectives and description of the study protocol**

This research was set up by the Catholic University of Leuven and the AMC (Amsterdam Medical Centre). Below, we always call AMC the ‘sponsor’. We invite you to participate in a clinical intervention study on psychotherapy treatment (Attachment Based Family Therapy; ABFT) in approximately 138 participants, of whom about 69 in Belgium.

Attachment Based Family Therapy is an intervention developed in America with the goal of reducing suicidal thoughts and behaviors by identifying and repairing fractures in the parent-child relationship, making it easier for young people to seek help during stress and thus less likely to desire suicide. At ABFT, the young adult and parent(s)/caregiver(s) receive approximately 16 treatment sessions (this may be slightly less, or slightly more), spread over 16 weeks.

Suicidal thoughts among young adults are common. Standard treatment for these complaints is the initiation of psychological treatment, possibly in combination with medication. There is now evidence that Attachment Based Family Therapy is successful in reducing suicidal thoughts in young adults.

The ABFT will be compared with other treatments offered in the Netherlands and Belgium. These can be different treatments. Attachment Based Family Therapy is seen as one of the standard treatments for suicide cases in the Netherlands and Belgium. However, it has never been investigated in the Netherlands or Belgium whether ABFT is more or as successful as the other treatments. This is what we are going to do in this REPAIR project. REPAIR stands for REbuilding Parent young Adult Interpersonal Relationships. Researchers, these can be researchers, psychologists and psychiatrists, conduct the research in different institutions in Belgium and the Netherlands.

*Are you suitable to participate?*

In order for you to participate in the study, it is important that your child is a suitable candidate. Thus, we first want to know if your child is suitable to participate. Therefore, the researcher will do an interview of about 15 minutes with your child, after the information and consent form has been signed.

In order to participate, it is important to know what psychological symptoms your child is experiencing and to what extent. For this purpose, a brief preliminary investigation will take place with your child, after the information and consent form has been signed. The preliminary examination can take place face to face, by telephone or via video call. Your child can indicate his/her preference. If after this preliminary examination it appears that your child can participate in the study and you and your child indicate in this information and consent letter that you wish to participate, your child and you, parent/guardian, will be given a number of questionnaires. The questionnaires will be about your child's symptoms/symptoms, attachment, family functioning or the relationship with the therapist in the study (during the period of therapy). The time it will take to complete the questionnaires can be found in the table p. 4.

To participate in the study, your child must:

a) be between 18 and 25 years old;

b) have a score of 31 or above on the SIQ-JR (This is the Suicidal Ideation Questionnaire, a questionnaire to estimate the degree of suicidality in a given person; when the score is 31 or more, we speak of suicidality) ;

c) have at least one parent or caregiver participating in the assessment and treatment. This can be a biological parent, stepparent, grandparent, other relative or foster parent.

Exclusion criteria are (criteria that result in your inability to participate in the study):

a) other primary disorders:severe alcohol or cannabis use disorder, all other substances: moderate or severe substance use disorder, severe conduct disorder, evidence of psychotic features or prior psychosis ;

b) severe cognitive impairment (e.g. mental retardation, severe developmental disorders) as evidenced by educational records, parental reports and/or clinical impressions;

c) other circumstances that may affect participation (e.g. serious medical condition, relocation).

**Course of the study**

*How long does the investigation /study take?*

Your participation in the study will take approximately 17 months.

*Are you suitable to participate?*

In order for you to participate in the study, it is important that your child is a suitable candidate. Thus, we first want to know if your child is suitable to participate. Therefore, the researcher will do an interview of about 15 minutes with your child, after the information and consent form has been signed.

In order to participate, it is important to know what psychological symptoms your child is experiencing and to what extent. For this purpose, a brief preliminary investigation will take place with your child, after the information and consent form has been signed. The preliminary examination can take place face to face, by telephone or via video call. Your child can indicate his/her preference. If after this preliminary examination it appears that your child can participate in the study and you and your child indicate in this information and consent letter that you wish to participate, your child and you, parent/guardian, will be given a number of questionnaires.

*Step 2: the treatment*

As part of the study, your child will receive treatment for his/her suicidal thoughts and additional symptoms. This treatment will be followed up by the research team for 5 months. If treatment can be completed after these 5 months, it will be completed. If the clinician/therapist deems it necessary that further treatment is needed, either the treatment will be continued or a suitable next treatment will be sought so that your child does not simply fall without treatment.

For this research we make 2 groups:

• Group 1. The people in this group receive ABFT in addition to standard care. ABFT involves parents/caregivers. If your child is drawn in grade 1, you will also participate in the therapy sessions. In addition, you will also complete questionnaires as part of the study.

• Group 2. The people in this group receive standard care. Standard care is the care currently indicated for the treatment of suicidality in young adults. Usually this is some form of therapy, such as Cognitive Behavioral Therapy, combined with medication. Those in this group will not receive ABFT, so parents/caregivers will not be involved in therapy sessions. The parents/caregivers do participate in the study, which means that they will answer questionnaires.

A draw determines which treatment your child receives.

If your child is receiving ABFT treatment, we will ask for your and your child's permission to **record a the therapy sessions on video**. These video recordings will be used by the researchers **to verify that the ABFT treatment was given as intended**. In this video, we are looking primarily at the practitioner/therapist giving the therapy/treatment to verify that this therapist actually applied/gave the ABFT as it was intended. You and your child will be given the choice of whether or not to be filmed recognizably The video recordings are kept securely at your institution's location and on a secure drive (Research Drive of the AMC) of the client. Only the researchers will have access to these video recordings. After the researchers have reviewed the video recordings, they are promptly deleted. This ensures the protection of your and your child's identity. Under no circumstances will the video footage be shown to students for educational purposes and there will be no marketing or other commercial purposes. If you agree, please check this on the consent form. You and your child have the right at all times to indicate that you do not wish to participate in this study or no longer wish to be filmed during therapy. This will not affect any further participation in the study.

*Step 3: examinations and measurements*

We will send your child a questionnaire 5 times and you, parent(s) or caregiver(s), 4 times. The questions are about your child’s complaints and suicidal thoughts, attachment, family functioning or the relationship with the therapist in the study. It will take your child about 30 minutes to complete this questionnaire. The questionnaire for parent(s)/caregiver(s) takes about 10 minutes to be completed.

The researcher will also call your child a total of 4 times. Your child will then receive questions about his/her current suicidality complaints, or he/she will make an appointment with your child at the location of the institution where you are in treatment.. This conversation lasts about 30 minutes.

If your child and you decides to participate in the study and your child meets all the conditions for participation, you and your child will go through the following tests and studies:

Assessments for young adult

| **Appointment** | **Time** | **Activities** | **Duration** | **Location** |
| --- | --- | --- | --- | --- |
| Eligibility screening | After intake/after signing  Consent  form -  Informed Consent | Interview | 15 minutes | At home/ online, or at your treatment facility |
| Measurement 1 | After eligibility screening | Interview and questionnaires | 60 minutes | At home/ online, or at your treatment facility |
| Intervention | During first session, every two months, and last session | Questionnaire | 3 minutes per session | At home/ online, or at your treatment facility |
| Measurement 2 | After end of treatment | Interview and questionnaires | 60 minutes | At home/ online, or at your treatment facility |
| Measurement 3 | 3 months after treatment | Interview and questionnaires | 60 minutes | At home/ online, or at your treatment facility |
| Measurement 4 | 6 months after treatment | Questionnaires | 10 minutes | Online |
| Measurement 5 | 12 months after treatment | Interview and questionnaires | 60 minutes | At home/ online, or at your treatment facility |

Assessment for the parent(s)/caregiver(s)

| **Appointment** | **Time** | **Activities** | **Duration** | **Location** |
| --- | --- | --- | --- | --- |
| Measurement 1 | After eligibility screening of your child and informed consent | Questionnaires | 10 minutes | Online |
| Measurement 2 | After end of treatment | Questionnaires | 10 minutes | Online |
| Intervention | During first session, every two months, and last session | Questionnaire | 3 minutes per session | Online |
| Measurement 3 | 3 months after treatment | Questionnaires | 10 minutes | Online |
| Measurement 4 | 12 months after treatment | Questionnaires | 10 minutes | Online |

**Risks and inconveniences**

**What side effects, adverse effects or discomforts can you suffer from?**

There are no major risks associated with ABFT and standard care. However, it is possible, however, that completing the questionnaires may cause some discomfort to you and/or your child and thus may be emotionally taxing. All study participants will receive a handout if they are included, which lists help sites such as the helpline/suicide line (1813 in Belgium). Participants can also contact their practitioner/therapist in the study at any time.

Another disadvantage of the study is that it takes time from your child and you, parent/caregiver. The appointments that take place as part of this study will be scheduled according to your and your child's preferences as much as possible.

Possible side effects of your child's standard treatment:

- Psychological treatment: there are no major risks associated with psychological treatment. However, the therapy may be emotionally taxing.
- Medication: see package insert of the medication your child is taking for specification of side effects.

**What are the advantages and disadvantages of participating in the study?**

Participating in the research can have advantages and disadvantages. Below we list them. Think about this carefully, and talk about it with others.

The treatment ABFT can further reduce your child’s suicidal thoughts and additional complaints, but that is not certain. At any time during this examination, these complaints may come back or worsen. If symptoms worsen or a crisis situation occurs, practitioners/therapists and investigators always consult the crisis protocol, which describes procedures on what to do in a crisis situation. In this way, appropriate care will always be offered.

Participating in the study can have these disadvantages:

- Your child may experience the side effects or adverse effects described above. You, too, might find that completing the questionnaires is emotionally taxing.
- Participating in the study will cost you and your child extra time.
- You and your child must adhere to the agreements associated with the investigation.

*Do you or your child not want to participate?*

It is your and your child’s choice whether or not you wish to participate in this study. Do you and/or your child not wish to participate? Then your child will receive the standard treatment for his/her suicidal thoughts. Your practitioner/therapist can tell you more about the available options for treatment and about their pros and cons.

**When does the study end?**

The investigator will let you and your child know if there is any new information about the study that is important to you and your child. The investigator will then ask you if you want to continue to take part.

In the following situations, the study will stop for you:

- All check-ups according to the schedule are finished.
- The end of the study has been reached.
- You want to stop participating in the study. You can stop at any time. Report this to the investigator immediately. You do not have to explain why you want to stop. Your child will continue to receive treatment for his/her suicidal thoughts and co-morbid symptoms. The investigator will still invite your child for a follow-up check.
- The investigator thinks it is better for you to stop. The investigator will still invite your child for a follow-up check.
- One of the following authorities decides that the study should stop:
  - AMC (prof. Bockting), or
  - the government, or
  - the Medical Ethics Review Committee assessing the study

*What happens if you stop participating in the study?*

If you withdraw your consent to participate in the study, the coded data already collected before your withdrawal will be retained. This will ensure the validity of the study. No new data will be transmitted to the sponsor.

The entire study ends when all the participants have finished their treatments and check-ups.

**Treatment after discontinuation of the study**

In all situations where participation in the study is stopped, but also when the study is completed as planned, the researcher/therapist will examine your health and prescribe you the best treatment available. So you will still receive your treatment for suicidal thoughts and additional complaints. The researcher/therapist can arrange one or more checks for your safety.

**What happens after the study?**

*Will you receive the results of the study?*

After processing all the data, about a year after your participation, the researcher will let you know what the most important results of the study are.

**If you participate in this study, we ask you the following:**

We would like the research to go well. That is why we make the following agreements with you:

- You and your child follow the treatment as agreed with your practitioner/therapist.
- You come to every appointment, in case it is asked, together with your child.
- You contact the researcher in these situations:
  - You and/or your child will be hospitalized or treated.
  - You and/or your child suddenly have problems with your health.
  - You and/or your child no longer want to participate in the study.
  - Your and/or your child’s phone number, address or email address will change.

**You should also know that:**

- in order to participate in this clinical trial and for you and your child's safety, you and your child must agree that the investigator may inform the various physicians, therapists or other specialists involved in treatment about your child's and your participation in this clinical trial. In the event of increasing suicidal thoughts in your child, crisis situations or other situations where it is deemed necessary, we may then contact your and/or your child's primary care physician and/or treating specialist, for example, about the status of current treatment.

**What will happen to your data?**

*What data do we store?*

We store the following data:

- your name

- your gender

- your address

- your date of birth

- information about your health

- (medical) information that we collect during the study

*Why do we collect, use and store your data?*

We collect, use, and store your data to answer the questions of this study and to be able to publish the results.

*How do we protect your privacy?*

To protect your privacy, your data will be given a code. We only use this code for your personal data. We keep the key to the code in a safe place at the facility/institution where you are being treated at, so only at the participating site . When we process your data, we always use only that code. Your name or personal data will never appear in any report or publication about this research. The coded data will be securely shared with the statistical experts, who are associated with our study, to analyze the (cost)-effectiveness of the treatment.

*Who can see your data?*

In order to control the quality of the study, it is possible that your non-coded personal data or information from your medical record relevant to this study may be inspected by people other than the study staff. This inspection is done under the supervision of the researcher and these people are bound by professional secrecy or through a confidentiality agreement. They may be:

- personnel designated by the client (MONITORS and AUDITORS) and people or organizations providing services to or working with the client. However, they will never disclose your name and con-tact information to the client.

- inspectors from competent health authorities around the world
- an independent audit group
- persons appointed by the Ethics Committee

*How long do we keep your data?*

After the study ends, your coded data will be kept for at least 25 years (Ref. 1) to ensure the validity of the study. This will be the case even if you stop participating in the study early. The data may still be viewable in the context of an audit or inspection by the government or other authorities in connection with the treatment examined. As soon as this is no longer necessary, we will destroy your data.

By giving permission to participate in this study, you also agree that your coded data from this study can be used by the grant providers (BeNeFIT call funders, being KCE in Belgium and ZonMw in the Netherlands) or similar public health research institutes in Europe for further analyses. For example, to determine whether one of the treatments examined offers an additional value.

Under no circumstances will the researchers carrying out the additional analyses see your identity and all researchers are bound by professional secrecy.

*Can we use your data for other research?*

At the end of this examination, your data may also be important for **other scientific research** on suicidal thoughts and treatments. This study remains within the context of the current clinical trial and thus aims to better understand symptoms of your child, their treatment and response to this treatment. For this purpose, your data will be stored with at the sponsor site for 25 years.

*What happens with accidental finds?*

A result found by chance during the study and on top of the objectives is called an accidental find. If this result may be important to your health or that of your blood relatives, the sponsor will inform the investigator. With your consent, the researcher will inform you and your practitioner/therapist/treating physician of your results and the possible consequences. If necessary, the researcher and/or practitioner/therapist/treating physician will advise you on what to do.

You agree or disagree to be informed by checking the appropriate box on page 10.

*What happens to your data if you no longer wish to participate in the study?*

You can withdraw your participation in the study at any time. But please note: do you decide to stop the study, and have researchers already collected data for a study? Then they may still use this data.

*Would you like to know more about your privacy?*

- Would you like to know more about your rights when processing personal data? Then take a look at [www.gegevensbeschermingsautoriteit.be](http://www.gegevensbeschermingsautoriteit.be)
- Do you have questions about your rights? Or do you have a complaint about the processing of your personal data? Please contact the person responsible for processing your personal data. For your research, that is:
  - KU Leuven. See Appendix A for contact details, and website.
- If you have any complaints about the processing of your personal data, we recommend that you first discuss them with the research team. You can also report this to the Belgian supervisory authority which monitors compliance with the basic principles of the protection of personal data (see Appendix A).

*Where can you find more information about the study?*

You can find more information about the study on the following website: <https://repairstudy.com>

**Will you receive compensation if you participate in the study?**

The tests and treatment for the study will not cost you anything. Your child will receive a voucher of €15,- per assessment. The four assessments for which your child will receive a voucher are the initial assessments, immediately after the treatment, and the ones 3 and 12 months after the treatment. Your child will receive €5.- for the online questionnaire 6 months after treatment. In total, your child can earn €65,- by participating in all 5 assessments. This does not apply to parent(s)/caregiver(s). Parent(s)/caregiver(s) receive €8 per assessment, which amounts to a maximum of €32,- in total. If you or your child stop before the study is finished, the compensation you and/or your child receive will be less.

### Are you insured during the study?

Any participation in a clinical study involves a risk, however small it is. Even if there is no fault, the sponsor accepts responsibility for damage caused to the participant (or in the event of death, his/her dependants) and directly or indirectly linked to his/her participation in the study. The sponsor has taken out insurance for this responsibility^[[8]](#footnote-8)^.

You are therefore asked to report any new health problem to the investigator before consulting another therapist, taking any other medication or receiving any other therapeutic treatment. If, for any reason, you or your child consult another therapist during this clinical study, you must inform him/her that you and your child are taking part in a clinical study. This could be important in establishing a diagnosis and treating your child’s complaints.

If the investigator believes that a link with the study is possible (the insurance does not cover the natural progression of your disease or the known side effects of your normal treatment), he/she will inform the study sponsor, which will initiate the declaration procedure to the insurance company. The latter will appoint an expert - if it considers it necessary - to assess whether there is a link between your new health problems and the study.

In the event of disagreement either with the investigator or with the expert appointed by the insurance company and also whenever you feel it is appropriate, you or - in case of death - your dependants may bring proceedings against the insurer directly in Belgium (name of insurance company, policy number, contact).

The law provides that the insurer may be summoned to appear either before the judge of the location where the event giving rise to the damage occurred, or before the judge of your domicile, or before the judge of the insurer’s registered offices.

**Contact**

If you need further information, but also if you have problems or concerns, you can contact the investigator (Surname, First name) or a member of his/her research team (Surname, First name) on the following telephone number (xx / xxx-xx-yy).

In case of emergency, you can contact XX on the following telephone number XX.

If you have any questions relating to your rights as a participant in a study, you can contact the ethics committee on this telephone number:+32 16 34 86 00 (weekdays from 10 a.m. to 11 a.m.).

**How do you give consent for the study?**

You can first carefully think about this study. Then you tell the investigator of the KU Leuven (independent of the health care provider) if you understand the information and if you want to take part or not. If you and your child want to take part, fill in the consent form that you can find with this information sheet. You, your child, and the investigator will get a signed version of this consent form.

Thank you for your time.

**REPAIR project: Treating suicidality in young adults: a study on Attachment Based Family Therapy**

# II Informed consent

**Parent(s) / caregiver(s)**

I declare that I have been informed of the nature of the study, its purpose, its duration, any risks and benefits and what is expected of me. I have taken note of the information document and the appendices to this document.

I have had sufficient time to think about it and discuss it with a person of my choice, such as my GP or a member of my family.

I have had the opportunity to ask any questions that came to mind and have obtained a satisfactory response to my questions.

I understand that my participation in this study is voluntary and that I am free to end my participation in this study without this affecting my relationship with the therapeutic team in charge of my health.

I understand that data about me will be collected throughout my participation in this study and that the investigator and the sponsor of the study will guarantee the confidentiality of these data in accordance with applicable European and Belgian legislation.

I agree to my personal data being processed as described in the section dealing with confidentiality guarantees (appendix B). I also consent to these data being transferred to and processed in countries other than Belgium.

I agree to the GP or other specialists in charge of the treatment of my health being informed of my participation in this clinical study.

I give consent to give my doctor, specialist or therapist information about accidental discoveries that may occur during the study that are important for my health.

I know that some people will be able to see all of my data to review the study. These people are mentioned in this information sheet. I give consent to let them see my data for this review.

I have received a copy of the information provided to the participant and the informed consent.

Please tick yes or no in the table below.

| I give permission for our therapy sessions to be videotaped (where I have the choice of being filmed recognizably or not on the video footage). | Yes ☐ | No  ☐ |
| --- | --- | --- |
| I give consent to ask me after this study if I want to participate in a follow-up study. | Yes ☐ | No  ☐ |
| I agree that in case of an accidental finding, the researcher will notify the practitioner/therapist/treating physician and myself of this and the possible consequences. | Yes ☐ | No  ☐ |
| I consent to my e-mail address being used in the electronic data system to send me the questionnaires and that my telephone number and address data are used in case this is necessary. | Yes ☐ | No  ☐ |

Surname, first name, date and signature of the volunteer.

**Investigator**

I, the undersigned, [surname, first name] investigator/clinical study assistant, confirm that I have verbally provided the necessary information about the study and have given the participant a copy of the information document.

I confirm that no pressure was applied to persuade the patient to agree to take part in the study and that I am willing to answer any additional questions if required.

I confirm that I operate in accordance with the ethical principles set out in the latest version of the “Helsinki Declaration”, the “Good Clinical Practices” and the Belgian Law of 7 May 2004 related to experiments on humans.

Surname, first name, date and signature

of the investigator’s representative

**REPAIR project: Treating suicidality in young adults: a study on Attachment Based Family Therapy**

# III Supplementary information

**Appendix A: contact information of Amsterdam UMC**

**Contact details of the participating site**

Principal Investigator at the site:

Coordinator at site:

Therapists at the site:

**Principal investigator:**

Professor Claudi Bockting

Meibergdreef 9

1105 AZ Amsterdam

Tel: +3120-8913600

E-mail: [c.l.bockting@amsterdamumc.nl](mailto:c.l.bockting@amsterdamumc.nl)

**Researchers**:

Dr. Nadia van der Spek

Meibergdreef 9

1105 AZ Amsterdam

Tel: +3120-8913600

E-mail: [n.vanderspek@amsterdamumc.nl](mailto:n.vanderspek@amsterdamumc.nl)

Professor Guy Bosmans

KU Leuven

Tiensestraat 102, 3000 Leuven, Belgium

Department of clinical psychology

Tel: +3216326187

E-mail: [guy.bosmans@kuleuven.be](mailto:guy.bosmans@kuleuven.be)

Luana Gavan

Meibergdreef 5

1105AZ Amsterdam

Tel: +3120-8913671

Email: [l.gavan@amsterdamumc.nl](mailto:l.gavan@amsterdamumc.nl)

Nele Bergers

KU Leuven

Tiensestraat 102, 3000 Leuven, Belgium

Department of clinical psychology

Tel: +3216326187

Email: [nele.bergers@kuleuven.be](mailto:nele.bergers@kuleuven.be)

**Complaints with regard to the processing of personal data**

The Belgian supervisory authority is called:

Gegevensbeschermingsautoriteit (GBA)

Drukpersstraat 35,

1000 Brussel

Tel. +32 2 274 48 00

e-mail: contact(at)apd-gba.be

Website: [www.gegevensbeschermingsautoriteit.be](http://www.gegevensbeschermingsautoriteit.be)

**Appendix B: Supplementary information on the protection and the rights of the participant in a clinical study**

### *Ethics Committee*

This study has been reviewed by an independent Ethics Committee, namely the Ethics Committee of [Name of EC], which has issued a favourable opinion [*after consulting with the Ethics Committees of each centre where this trial will be conducted*]. It is the task of the Ethics Committees to protect people who take part in a clinical trial. They make sure that your rights as a patient and as a participant in a clinical study are respected, that based on current knowledge, the balance^[[9]](#footnote-9)^ between risks and benefits remains favourable to the participants, that the study is scientifically relevant and ethical.
You should not under any circumstances take the favourable opinion of the Ethics Committee as an incentive to take part in this study.

### *Voluntary participation*

Before signing, do not hesitate to ask any questions you feel are appropriate. Take the time to discuss matters with a trusted person if you so wish.

Your participation in the study is voluntary and must remain free of any coercion: this means that you have the right not to take part in the study or to withdraw without giving a reason, even if you previously agreed to take part. Your decision will not affect your relationship with the investigator or the quality of your child’s future therapeutic care.

However, it is advisable for your child’s safety to inform the investigator if you have decided to stop taking part in the study.

If you agree to take part, you will sign the informed consent form. The investigator will also sign this form to confirm that he/she has provided you with the necessary information about the study. You will receive a copy of the form.

### *Costs associated with your participation*

### If you decide to participate in this study, this will not entail any additional costs for you, your child or your insurance company. The visits and procedures indicated in the description of the conduct of the investigation on pages x1 to x2/y as specific to the investigation are the responsibility of the sponsor. Only costs related to usual medical/psychological performance in your clinical situation can be charged to you. So you and/or your child only pay the costs that you would normally pay for a treatment.

### *Guarantee of confidentiality*

Your participation in the study means that you agree to the investigator collecting data about you and to the study sponsor using these data for research purposes and in connection with scientific and medical publications.

The processing of your personal data is necessary to achieve the scientific research purposes as set out herein. We would like to inform you that the necessity of the processing for the conduct of scientific research as a task of public interest constitutes the lawful basis on which we process your information in the context of the study in which you are participating.

Your data will be processed in accordance with the European General Data Protection Regulation (GDPR) and with the Belgian legislation on the protection of natural persons with regard to the processing of personal data. Academisch Medisch Centrum, Amsterdam shall act as data controller for your data.

The legal basis for processing your data is the public interest. This means that the research will lead to an increase in knowledge and insight that will benefit society (directly or indirectly).

You are entitled to ask the investigator what data are being collected about you and what is their use in connection with the study. This data concerns you and your child’s current clinical situation but also some of you and your child’s background, the results of examinations carried out within the context of care of your child’s health in accordance with the current standards and obviously the results of examinations required by the protocol. You and your child have the right to inspect these data and correct them if they are incorrect^[[10]](#footnote-10)^.

The investigator has a duty of confidentiality vis-à-vis the data collected.

This means that he/she undertakes not only never to reveal your name in the context of a publication or conference but also that he/she will encode (your identity will be replaced by an ID code in the study) your data before sending them to the manager of the database of collected data (controller: AMC, location of the database: on the AMC's secured L or G drive).

The therapists at the treating sites and investigator and his/her team will therefore be the only ones to be able to establish a link between the data transmitted throughout the study and your medical records^[[11]](#footnote-11)^.

The personal data transmitted will not contain any combination of elements that might allow you or your child to be identified^[[12]](#footnote-12)^.

For the study data manager designated by the sponsor, the data transmitted will not allow you or your child to be identified. The latter is responsible for collecting the data gathered by all investigators taking part in the study, processing them and protecting them in accordance with the requirements of the Belgian law on the protection of privacy.

To verify the quality of the study, it is possible that you and your child’s medical records will be examined by persons subject to professional secrecy and designated by the ethics committee, the sponsor of the study or an independent audit body. In any event, this examination of your medical records may only take place under the responsibility of the investigator and under the supervision of one of the collaborators designated by him/her.

The (encoded) study data will be able to be sent to Belgian or other regulatory authorities, to the relevant ethics committees, to other doctors and/or to organisations working in collaboration with the sponsor.

They will also be able to be sent to other sites of the sponsor in Belgium and in other countries where the standards in terms of the protection of personal data may be different or less stringent? As explained above, the transmitted data are encoded^[[13]](#footnote-13)^.

Your consent to take part in this study therefore also implies your consent to the use of your encoded medical data for the purposes described in this information form and to their transmission to the aforementioned people and authorities.

The sponsor will use the data collected within the context of the study in which you are taking part, but would also like to be able to use them in connection with other research concerning the same psychological difficulties as your child’s. Any use of your data outside the context described in this document is only possible with the approval of the ethics committee.

If you withdraw your consent to take part in the study, to guarantee the validity of the research, the data encoded up to the point at which you withdraw will be retained. No new data may be sent to the sponsor.

If you have any questions relating to how your data are being processed, you may contact the investigator. The data protection officer in your hospital can be contacted as well: DPO - UZ Leuven, Herestraat 49, 3000 Leuven, e-mail [dpo@uzleuven.be](mailto:dpo@uzleuven.be).

Finally, if you have a complaint concerning the processing of your data, you can contact the Belgian supervisory authority who ensures that privacy is respected when personal data are processed.

The Belgian supervisory authority is called:

Data Protection Authority (DPA)

Drukpersstraat 35,

1000 Brussels

Tel. +32 2 274 48 00

e-mail: contact@apd-gba.be

Website: <https://www.dataprotectionauthority.be>

### *Insurance*

Any participation in a clinical study involves a risk, however small it is. Even if there is no fault, the sponsor accepts responsibility for damage caused to the participant (or in the event of death, his/her dependants) and directly or indirectly linked to his/her participation in the study. The sponsor has taken out insurance for this responsibility^[[14]](#footnote-14)^.

You and your child are therefore asked to report any new health problem to the investigator before consulting another therapist, taking any other medication or receiving any other therapeutic treatment. If, for any reason, you and/or your child consult another therapist during this clinical study, you and your child must inform him/her that you are taking part in a clinical study. This could be important in establishing a diagnosis and treating your child’s complaints.

If the investigator believes that a link with the study is possible (the insurance does not cover the natural progression of your disease or the known side effects of your normal treatment), he/she will inform the study sponsor, which will initiate the declaration procedure to the insurance company. The latter will appoint an expert - if it considers it necessary - to assess whether there is a link between your new health problems and the study.

In the event of disagreement either with the investigator or with the expert appointed by the insurance company and also whenever you feel it is appropriate, you or - in case of death - your dependants may bring proceedings against the insurer directly in Belgium (name of insurance company, policy number, contact).

The law provides that the insurer may be summoned to appear either before the judge of the location where the event giving rise to the damage occurred, or before the judge of your domicile, or before the judge of the insurer’s registered offices.

Informed Consent Letter Young Adults – the Netherlands

**Subject information for participation in medical research**

**REPAIR study**

*Targeting suicidality in young adults: a randomized, controlled pragmatic, multi-centre trial evaluating the (cost)-effectiveness of Attachment Based Family Therapy compared to Treatment as Usual*

Dear Sir/Madam,

With this letter, we would like to ask you to take part in a medical study. Participation is voluntary. You have received this letter because you have suicidal thoughts for which you seek or receive treatment.

You can read about the medical study in this information sheet, what it means for you, and what the pros and cons are. It is a lot of information. Can you please read the information and decide if you want to take part in this study? If you want to take part, please complete the form in Appendix D.

**Ask your questions**

You can make your decision based on the information in this information sheet. We also suggest that you do the following:

- If you have any questions, please contact the investigator who gave you this information.

- Talk to your partner, family or friends about this study.

- If you have any questions, please contact the independent expert, Dr. Huibert Burger.

- Read the information on [Medical research: information for human subjects | Leaflet | Government.nl](https://www.government.nl/documents/leaflets/2020/06/03/medical-research-information-for-human-subjects)
 and [Human subjects | The Central Committee on Research Involving Human Subjects (ccmo.nl)](https://english.ccmo.nl/human-subjects).

1. **General information**

The AMC and the Catholic University of Leuven have set up this study. Below, we always refer to AMC as the ‘sponsor’. Investigators, which includes researchers, psychologists, and psychiatrists, are conducting this research in various institutions in the Netherlands and Belgium. This study requires 138 subjects in total from both countries. In the Netherlands, it is expected that 69 people will take part. The Medical Ethics Review Committee of the Amsterdam UMC has approved this study.

**2. What is the purpose of the study?**

In this study, we investigate whether Attachment Based Family Therapy (ABFT) is successful in reducing suicidal thoughts in young adults. This treatment will be compared to other standard care that is offered in the Netherlands and Belgium.

1. **What is the background of the study?**

Suicidal thoughts are common among young adults. Standard treatment for these complaints is the initiation of treatment, possibly in combination with medication. There is now evidence that ABFT is successful in reducing suicidal thoughts in young adults.

ABFT is an intervention from America aimed at reducing suicidal thoughts by helping young people to share their problems with their parent(s)/caregiver(s) with confidence. Additionally, ABFT may help parent(s)/caregiver(s) support their child in a way that makes their child feel understood and heard. The young adults and parent(s)/caregiver(s) receive approximately 16 ABFT treatment sessions.

The ABFT will be compared to other standard treatments offered in the Netherlands and Belgium. These can be different treatments. ABFT is considered one of the standard treatments for suicidal thoughts in the Netherlands and Belgium. However, in the Netherlands and Belgium it has never been studied whether ABFT is more successful or as successful as other treatments. That is what we will investigate with this REPAIR study.

1. **What happens during the study?**

*How long will the study take?*

Are you taking part in the study? It will take about 17 months in total.

*Step 1: are you eligible to take part?*

First, we want to know if you are eligible to take part. That is the reason that the investigator is doing an interview of approximately 15 minutes.

To participate, it is important to know which psychological complaints you are experiencing and to what extent. A short preliminary investigation will take place to determine this, which will take place via telephone or video call. You can indicate your preference. If it appears after this survey that you can participate in the survey, you and your parent(s)/caregiver(s) will receive questionnaires. See Step 3 and Appendix C for an overview of the questionnaires.

*Step 2: the treatment*

You will receive treatment for your suicidal thoughts for approximately 5 months.

For this study, we will have 2 groups:

- Group 1. The people in this group will get ABFT in addition to care as usual.
- Group 2. The people in this group will get care as usual.

A draw will decide which treatment you are given.

During the treatment, we will record the sessions with permission from you and your parent(s)/guardian(s). We only do this with written permission.

*Step 3: study and measurements*

We will send you questionnaires 5 times and your parent(s)/caregiver(s) 4 times. The questions are about suicidal thoughts and related complaints. It will take around 30 minutes to complete this questionnaire. The questionnaire for your parent(s)/caregiver(s) will take around 10 minutes to complete.

An investigator will interview you 4 times in person or over the phone. You will receive questions regarding suicidal thoughts and related complaints. This interview will take approximately 30 minutes. During this interview the Secure Base Script task (Attachment Interview) will also be administered; it is necessary to make an audio or video recording of the Secure Base Script task as part of the data collection process, in order to be able to score and analyse this task.

In Appendix C you can see all the assessments at each moment.

1. **What agreements do we make with you?**

We want the study to go well. That is why we want to make the following agreements with you:

- You and your parent(s)/caregiver(s) follow the treatment in the way the investigator explained to you.
- You go to every appointment and when it is requested from you with your parent(s)/caregiver(s).
- You should contact the investigator in these situations:
  - You and or your parent(s)/caregiver(s) are hospitalised or receiving treatment at a hospital.
  - You and or your parent(s)/caregiver(s) suddenly have problems with your health.
  - You and or your parent(s)/caregiver(s) no longer want to take part in the study.
  - Your and or your parent(s)/caregiver(s) telephone number, address or email address changes.

1. **What side effects, adverse effects, or discomforts could you experience?**

There are no risks associated with ABFT and standard care. However, a disadvantage of the study is that it takes up your and your parent’s/parents’/caregiver’s/caregivers’ time. The appointments that take place in the context of this study will be scheduled according to your preference as much as possible. The investigations are all painless. There are no disadvantages or risks associated with completing the questionnaires about your complaints. However, the questions may be emotionally taxing for you.

1. **What are the pros and cons if you take part in the study?**

Taking part in the study can have pros and cons. We will list them below. Consider this list carefully and discuss it with other people.

The treatment ABFT may reduce your suicidal thoughts and co-morbid symptoms, but that is not guaranteed. Your symptoms may come back or get worse at any time during this study.

Taking part in the study can have these cons:

- - You may experience side effects or adverse effects, as described in Section 6.
  - Taking part in the study will take up more of your time.
  - You have to comply with the agreements of this study.

*Do you or your parent/caregiver not wish to participate in the study?*

It is your choice whether or not you wish to participate in this study. Do you not wish to participate? Then you will receive another standard treatment for your suicidal thoughts. Your therapist can tell you more about the available options for treatment and about their pros and cons.

1. **When does the study end?**

The investigator will let you know if there is any new information about the study that is important to you. The investigator will then ask you if you want to continue to take part.

In the following situations, the study will stop for you:

- All check-ups according to the schedule are finished.
- The end of the study has been reached.
- You want to stop participating in the study. You can stop at any time. Report this to the investigator immediately. You do not have to explain why you want to stop. You will continue to receive treatment for your suicidal thoughts and co-morbid symptoms. The investigator will still invite you for a follow-up check.
- The investigator thinks it is better for you to stop. The investigator will still invite you for a follow-up check.
- One of the following authorities decides that the study should stop:
  - AMC (prof. Bockting), or
  - the government, or
  - the Medical Ethics Review Committee assessing the study

*What happens if you stop participating in the study?*

The investigators use the data that have been collected up to the moment that you decided to stop participating in the study.

The entire study ends when all the participants have finished their treatments and check-ups.

1. **What happens after the study has ended?**

*Will you get the results of the study?*

About a year after you take part in the study, the investigator will inform you about the most important results of the study.

1. **What will happen to your data?**

Are you and your parent(s)/caregiver(s) taking part in the study? Then you and your parent(s)/caregiver(s) also consent to us collecting, using, and storing your data.

*What data do we store?*

We store the following data:

- your name

- your gender

- your address

- your date of birth

- information from your parent(s)/caregiver(s)

- information about your health

- (medical) information that we collect during the study

*Why do we collect, use and store your data?*

We collect, use, and store your data to answer the questions of this study and to be able to publish the results.

*How do we protect your privacy?*

To protect your privacy, your data will be given a code. We only use this code for your personal data. We keep the key to the code in a safe place at the sponsor site. When we process your data, we always use only that code. Your name or personal data will never appear in any report or publication about this research. The coded data will be securely shared with the statistical experts, who are associated with our study, to analyse the (cost)-effectiveness of the treatment. The coded data can also be requested by the subsidy providers (BeNeFIT call financiers: KCE in Belgium and ZonMw in the Netherlands) for further analyses.

*Who can see your data?*

Some people can see your name and other personal information without a code. These are people checking whether the investigators are carrying out the study properly and reliably. These people can access your data:

- Members of the committee that keeps an eye on the safety of the study.
- An auditor who works for the sponsor.
- National and international supervisory authorities. For example, the Healthcare and Youth Inspectorate.

These people will keep your information confidential. We ask you to give permission for this access.

*For how long do we store your data?*

We store your data at the sponsor site for 15 years.

The data will be stored for 15 years in order to be able to make new assessments related to this study in the course of this study. If no longer needed, we will destroy your data.

*Can we use your data for other research?*

Your data may also be important after this study for other medical research on suicidal thoughts and treatments. For this purpose, your data will be stored at the sponsor site for 15 years. Please indicate in the consent form whether you agree to this. Do you not want to give your consent? Then you can still take part in this study. You will receive the same treatment.

*What happens if there are accidental discoveries?*

It is possible that during the study we discover something that is important to your health or to the health of your family members. In that case, the investigator will contact your current therapist or GP. You will then discuss what needs to be done with your doctor or therapist. By giving your consent on this form, you consent that your doctor or therapist will be informed.

*Can you withdraw your consent for the use of your data?*

You can withdraw your consent for the use of your data at any time. Please note: if you withdraw your consent, and the investigators have already collected data for research, they are still allowed to use this information.

*Do you want to know more about your privacy?*

- Do you want to know more about your rights when processing personal data? Visit [Autoriteit Persoonsgegevens |](https://www.autoriteitpersoonsgegevens.nl/en).
- Do you have any questions about your rights? Or do you have a complaint about the processing of your personal data? Please contact the person who is responsible for processing your personal data. At present, this is:
  - AMC see Appendix A for contact details, and website.
- If you have any complaints about the processing of your personal data, we recommend that you first discuss this with the research team. You can also contact the Data Protection Officer of Amsterdam UMC, location AMC. Or you can submit a complaint to the Dutch Data Protection Authority.

*Where can you find more information about the study?*

You can find more information about the study on the following website: <https://repairstudy.com>

1. **Will you receive compensation if you participate in the study?**

The tests and treatment for the study will not cost you anything. You will receive a voucher of €15,- per assessment. The four assessments for which you will receive a voucher are the initial assessments, immediately after the treatment, and the ones 3 and 12 months after the treatment. You will receive €5.- for the online questionnaire 6 months after treatment. In total, you can earn €65,- by participating in all 5 assessments. This does not apply to parent(s)/caregiver(s). Parent(s)/caregiver(s) receive €8 per assessment, which amounts to a maximum of €32,- in total. If you or your parent(s)/caregiver(s) stop before the study is finished, the compensation you receive will be less.

1. **Are you insured during the study?**

Insurance has been taken out for everyone who participates in this study. The insurance pays for damage caused by the investigation. But not for all damage. In appendix B you will find more information about the insurance and the exceptions. It also states to who you can report the damage.

1. **We will inform your GP and therapist**

The investigator will send your GP and therapist a letter or email to let them know that you are taking part in the study. This is for your safety. In the event of increasing suicidal thoughts or other crises, we can contact your general practitioner and/or therapist, for example, about the status of your current treatment.

1. **Do you have any questions?**

If you have any questions about this study, feel free to contact the research team. Would you like to get advice from someone who is independent of the study? In this case, you can contact Dr. Burger. He knows a lot about the study but is not a part of this study.

Do you have a complaint? Discuss it with the investigator or the doctor who is treating you. If you prefer not to do so, please visit the complaints officer or complaints committee of your hospital. Appendix A contains the contact information.

1. **How do you give consent for the study?**

You can first carefully think about this study. Then you tell the investigator of the treatment facility (independent of the health care provider) if you understand the information and if you want to take part or not. If you and your parent(s)/caregiver(s) want to take part, fill in the consent form that you can find with this information sheet. You, your parent(s)/caregiver(s), and the investigator will get a signed version of this consent form.

Thank you for your time.

**16. Appendices to this information**

A. Contact information <*to be adjusted per participating centre*>

B. Information about insurance

C. Overview of measurements

D. Consent form young adult

**Appendix A: contact information of AMC**

**Principal investigator:**

Professor Claudi Bockting

Meibergdreef 9

1105 AZ Amsterdam

Tel: +3120-8913600

E-mail: [c.l.bockting@amsterdamumc.nl](mailto:c.l.bockting@amsterdamumc.nl)

**Researchers**:

Dr. Nadia van der Spek

Meibergdreef 9

1105 AZ Amsterdam

Tel: +3120-8913600

E-mail: [n.vanderspek@amsterdamumc.nl](mailto:n.vanderspek@amsterdamumc.nl)

Professor Guy Bosmans

KU Leuven

Tiensestraat 102, 3000 Leuven, Belgium

Department of clinical psychology

Tel: +3216326187

E-mail: [guy.bosmans@kuleuven.be](mailto:guy.bosmans@kuleuven.be)

Luana Gavan

Meibergdreef 5

1105AZ Amsterdam

Tel: +3120-8913671

Email: l.gavan@amsterdamumc.nl

Nele Bergers

KU Leuven

Tiensestraat 102, 3000 Leuven, Belgium

Department of clinical psychology

Tel: +3216326187

Email: [nele.bergers@kuleuven.be](mailto:nele.bergers@kuleuven.be)

**Independent doctor/expert:**

Dr. Huib Burger, MD

Universitair Medisch Centrum Groningen

Antonius Deusinglaan 1

9713 AV Groningen

Tel: +3150 361 6724

E-mail: [h.burger@umcg.nl](mailto:h.burger@umcg.nl)

**Complaints:**

Afdeling Patiëntenvoorlichting & Klachtenopvang, A0-404/406

Meibergdreef 5

1105 AZ Amsterdam

Tel: +3120-5663355

E-mail: [patientenvoorlichting@amc.nl](mailto:patientenvoorlichting@amc.nl)

**Data Protection Officer of the institution:**

E-mail: [privacy@amsterdamumc.nl](mailto:privacy@amsterdamumc.nl)

**For more information about your rights:**

Afdeling Patiëntenvoorlichting & Klachtenopvang, A0-404/406

Meibergdreef 5

1105 AZ Amsterdam

Tel: 020-5663355

E-mail: [patientenvoorlichting@amc.nl](mailto:patientenvoorlichting@amc.nl)

**Appendix B: Information about the insurance**

The AMC has taken out insurance for everyone participating in this study. The insurance covers damage caused by participation in the study. This applies to damage during the research or within four years after its end. You must report damage to the insurer within those four years.

The insurance does not cover all damage. At the bottom of this text, you will find a brief description of which damage is not covered.

These provisions are contained in the Decision on compulsory insurance for medical research involving human subjects. This decision can be found on www.ccmo.nl, the website of the Central Committee on research involving human subjects (see 'Library' and then 'Laws and regulations').

In the event of damage, you can contact the insurer [or claims representative] directly.

| The insurer of the research is:  Name insurer: Centramed B.A.  Adres insurer: Postbus 7374  2701 AJ Zoetermeer  Telephone: 070 301 70 70  E-mail: info@centramed.nl  Polisnumber: 624.528.303 |
| --- |

The insurance offers a cover of € 650.000 per test subject with a maximum of

€ 5.000.000 for the entire research and € 7.500.000 for damage resulting from medical research involving human subjects, that is reported per insurance year.

The insurance does not cover the following damage:

- damage due to a risk about which you have been informed in the written information. This does not
 apply if the risk is more serious than anticipated or if the risk was very unlikely;

- damage to your health that would also have occurred if you had not participated in the study;

- damage due to not (completely) following directions or instructions;

- damage to your descendants as a result of a negative effect of the research on you or your
 descendants;

- damage caused by an existing treatment method in research into existing treatment methods.

Furthermore, the subject is requested to contact Professor Claudi Bockting in this regard (see attachment A for contact information).

**Appendix C: Overview of assessments**

Assessments for the subject

| **Appointment** | **Time** | **Activities** | **Duration** | **Location** |
| --- | --- | --- | --- | --- |
| Eligibility screening | After intake | Interview | 15 minutes | At home/ online, or at your treatment facility |
| Measurement 1 | After eligibility screening and informed consent | Interview and questionnaires | 60 minutes | At home/ online, or at your treatment facility |
| Intervention | During first session, every two months, and last session | Questionnaire | 3 minutes per session | At home/ online, or at your treatment facility |
| Measurement 2 | After end of treatment | Interview and questionnaires | 60 minutes | At home/ online, or at your treatment facility |
| Measurement 3 | 3 months after treatment | Interview and questionnaires | 60 minutes | At home/ online, or at your treatment facility |
| Measurement 4 | 6 months after treatment | Questionnaires | 10 minutes | Online |
| Measurement 5 | 12 months after treatment | Interview and questionnaires | 60 minutes | At home/ online, or at your treatment facility |

Assessments for the parent(s)/caregiver(s)

| **Appointment** | **Time** | **Activities** | **Duration** | **Location** |
| --- | --- | --- | --- | --- |
| Measurement 1 | After eligibility screening of your child and informed consent | Questionnaires | 10 minutes | Online |
| Measurement 2 | After end of treatment | Questionnaires | 10 minutes | Online |
| Intervention | During first session, every two months, and last session | Questionnaire | 3 minutes per session | Online |
| Measurement 3 | 3 months after treatment | Questionnaires | 10 minutes | Online |
| Measurement 4 | 12 months after treatment | Questionnaires | 10 minutes | Online |

**Appendix D: Informed consent form – subject**

This consent form belongs to REPAIR study

- I have read the information sheet. I was able to ask questions. My questions have been answered to satisfaction. I have had enough time to decide if I want to take part.
- I know that taking part is voluntary. I also know that at any time I can decide not to take part in the study. Or to stop taking part. I do not have to explain why.
- I give the investigator consent to inform my GP and therapist that I am taking part in this study.
- I give consent to request information from my GP and therapist about my suicidal thoughts and psychological symptoms.
- I give consent to give my doctor or therapist information about accidental discoveries that may occur during the study that are important for my health.
- I give consent to collect and use my data. The investigators only do this to answer the question of this study.
- I know that some people will be able to see all of my data to review the study. These people are mentioned in this information sheet. I give consent to let them see my data for this review.
- I know that an audio or video recording of the Secure Base Script task (Attachment Interview) is necessary as part of the data collection process, in order to be able to score and analyse this task. I give consent to audio or video record the section of the interview in which the Secure Base Script task (Attachment Interview) is administered.
- Please tick yes or no in the table below.

| I give consent to store my data to use for other research, as stated in the information sheet. | Yes ☐ | No  ☐ |
| --- | --- | --- |
| I give consent to videotape our therapy sessions. | Yes ☐ | No  ☐ |
|  |  |  |
| I give consent to ask me after this study if I want to participate in a follow-up study. | Yes ☐ | No  ☐ |

- I want to take part in this study.

My name is (subject): ………………………………..

Signature: ……………………… Date : __/__/__

-----------------------------------------------------------------------------------------------------------------

I declare that I have fully informed this subject about the study in question.

If any information becomes known during the study that could influence the subject's consent, I will let this subject know in good time.

Name investigator (or their representative): ........................

Signature:……………………… Date: __/__/__

-----------------------------------------------------------------------------------------------------------------

<if applicable>

Additional information was given by:

Name:………………………………..

Job title:………………………………

Signature:……………………… Date: __/__/__

-----------------------------------------------------------------------------------------------------------------

*The study subject will receive a complete information sheet and a signed version of the consent form.*

Informed Consent Letter Parent(s)/caregiver(s) – the Netherlands

**Subject information for participation in medical research**

**REPAIR study**

*Targeting suicidality in young adults: a randomized, controlled pragmatic, multi-centre trial evaluating the (cost)-effectiveness of Attachment Based Family Therapy compared to Treatment as Usual*

Dear Sir/Madam,

With this letter, we would like to ask you to take part in a medical study. Participation is voluntary. You have received this letter because your child has suicidal thoughts and seeks or receives treatment.

You can read about the medical study in this information sheet, what it means for you, and what the pros and cons are. It is a lot of information. Can you please read the information and decide if you want to take part in this study? If you want to take part, please complete the form in Appendix D.

**Ask your questions**

You can make your decision based on the information in this information sheet. We also suggest that you do the following:

- If you have any questions, please contact the investigator who gave you this information.

- Talk to your partner, family or friends about this study.

- If you have any questions, please contact the independent expert, Dr. Huibert Burger.

- Read the information on [Medical research: information for human subjects | Leaflet | Government.nl](https://www.government.nl/documents/leaflets/2020/06/03/medical-research-information-for-human-subjects)
 and [Human subjects | The Central Committee on Research Involving Human Subjects (ccmo.nl)](https://english.ccmo.nl/human-subjects).

1. **General information**

The AMC and the Catholic University of Leuven have set up this study. Below, we always refer to AMC as the ‘sponsor’. Investigators, which includes researchers, psychologists, and psychiatrists, are conducting this research in various institutions in the Netherlands and Belgium. This study requires 138 subjects in total from both countries. In the Netherlands, it is expected that 69 people will take part. The Medical Ethics Review Committee of the Amsterdam UMC has approved this study.

**2. What is the purpose of the study?**

In this study, we investigate whether Attachment Based Family Therapy (ABFT) is successful in reducing suicidal thoughts in young adults. This treatment will be compared to other standard care that is offered in the Netherlands and Belgium.

1. **What is the background of the study?**

Suicidal thoughts are common among young adults. Standard treatment for these complaints is the initiation of treatment, possibly in combination with medication. There is now evidence that ABFT is successful in reducing suicidal thoughts in young adults.

ABFT is an intervention from America aimed at reducing suicidal thoughts by helping young people to share their problems with their parent(s)/caregiver(s) with confidence. Additionally, ABFT may help parent(s)/caregiver (s) support their child in a way that makes their child feel understood and heard. The young adults and parent(s)/caregiver(s) receive approximately 16 ABFT treatment sessions.

The ABFT will be compared to other standard treatments offered in the Netherlands and Belgium. These can be different treatments. ABFT is considered one of the standard treatments for suicidal thoughts in the Netherlands and Belgium. However, in the Netherlands and Belgium it has never been studied whether ABFT is more successful or as successful as other treatments. That is what we will investigate with this REPAIR study.

1. **What happens during the study?**

*How long will the study take?*

Are you taking part in the study? It will take about 17 months in total.

*Step 1: are you eligible to take part?*

First, we want to know if your child is eligible to take part. That is the reason that the investigator is doing an interview of approximately 15 minutes.

To participate, it is important to know which psychological complaints your child is experiencing and to what extent. A short preliminary investigation will take place to determine this, which will take place via telephone or video call. Your child can indicate his/her/their preference. If it appears after this survey that you can participate in the survey, you and your child will receive questionnaires. See Step 3 and Appendix C for an overview of the questionnaires.

*Step 2: the treatment*

Your child will receive treatment for his/her/their suicidal thoughts for approximately 5 months.

For this study, we will have 2 groups:

- Group 1. The people in this group will get ABFT in addition to care as usual.
- Group 2. The people in this group will get care as usual.

A draw will decide which treatment your child is given.

During the treatment, we will record the sessions with permission from you and your child. We only do this with written permission.

*Step 3: study and measurements*

We will send you questionnaires 4 times and your child 5 times. The questions are about the suicidal thoughts and related complaints of your child. It will take around 30 minutes to complete this questionnaire for your child. The questionnaire for you will take around 10 minutes to complete.

An investigator will interview your child 4 times in person or over the phone. Your child will receive questions regarding suicidal thoughts and related complaints. This interview will take approximately 30 minutes.

In appendix C you can see all the assessments at each moment.

1. **What agreements do we make with you?**

We want the study to go well. That is why we want to make the following agreements with you:

- You and your child follow the treatment in the way the investigator explained to you.
- You attend the required appointment with your child when it is requested from you.
- You should contact the investigator in these situations:
  - You and or your child are hospitalised or receiving treatment at a hospital.
  - You and or your child suddenly have problems with your health.
  - You and or your child no longer want to take part in the study.
  - Your and or your child’s telephone number, address or email address changes.

1. **What side effects, adverse effects, or discomforts could you experience?**

There are no risks associated with ABFT and standard care. However, a disadvantage of the study is that it takes up your and your child’s time. The appointments that take place in the context of this study will be scheduled according to your preference as much as possible. The investigations are all painless. There are no disadvantages or risks associated with completing the questionnaires about your complaints. However, the questions may be emotionally taxing for your child.

1. **What are the pros and cons if you take part in the study?**

Taking part in the study can have pros and cons. We will list them below. Consider this list carefully and discuss it with other people.

The treatment ABFT may reduce your child’s suicidal thoughts and co-morbid symptoms, but that is not guaranteed. Your child’s symptoms may come back or get worse at any time during this study.

Taking part in the study can have these cons:

- - Your child may experience side effects or adverse effects, as described in Section 6.
  - Taking part in the study will take up more of your and your child’s time.
  - You and your child have to comply with the agreements of this study.

*Do you or your child not wish to participate in the study?*

It is your choice whether or not you wish to participate in this study. Do you not wish to participate? Then your child will receive another standard treatment for his or her suicidal thoughts. Your therapist can tell you more about the available options for treatment and about their pros and cons.

1. **When does the study end?**

The investigator will let you know if there is any new information about the study that is important to you. The investigator will then ask you if you want to continue to take part.

In the following situations, the study will stop for you:

- All check-ups according to the schedule are finished.
- The end of the study has been reached.
- You want to stop participating in the study. You can stop at any time. Report this to the investigator immediately. You do not have to explain why you want to stop. Your child will continue to receive treatment for their suicidal thoughts and co-morbid symptoms. The investigator will still invite your child for a follow-up check.
- The investigator thinks it is better for your child to stop. The investigator will still invite your child for a follow-up check.
- One of the following authorities decides that the study should stop:
  - AMC (prof. Bockting), or
  - the government, or
  - the Medical Ethics Review Committee assessing the study

*What happens if you stop participating in the study?*

The investigators use the data that have been collected up to the moment that you decided to stop participating in the study.

The entire study ends when all the participants have finished their treatments and check-ups.

1. **What happens after the study has ended?**

*Will you get the results of the study?*

About a year after you take part in the study, the investigator will inform you about the most important results of the study.

1. **What will happen to your data?**

Are you and your child taking part in the study? Then you and your child also consent to us collecting, using, and storing your data.

*What data do we store?*

We store the following data:

- your name

- your gender

- your address

- your date of birth

- information about your child

- information about your health

- (medical) information that we collect during the study

*Why do we collect, use and store your data?*

We collect, use, and store your data to answer the questions of this study and to be able to publish the results.

*How do we protect your privacy?*

To protect your privacy, your data will be given a code. We only use this code for your personal data. We keep the key to the code in a safe place at the sponsor site. When we process your data, we always use only that code. Your name or personal data will never appear in any report or publication about this research. The coded data will be securely shared with the statistical experts, who are associated with our study, to analyse the (cost)-effectiveness of the treatment. The coded data can also be requested by the subsidy providers (BeNeFIT call financiers: KCE in Belgium and ZonMw in the Netherlands) for further analyses.

*Who can see your data?*

Some people can see your name and other personal information without a code. These are people checking whether the investigators are carrying out the study properly and reliably. These people can access your data:

- Members of the committee that keeps an eye on the safety of the study.
- An auditor who works for the sponsor.
- National and international supervisory authorities. For example, the Healthcare and Youth Inspectorate.

These people will keep your information confidential. We ask you to give permission for this access.

*For how long do we store your data?*

We store your data at the sponsor site for 15 years.

The data will be stored for 15 years in order to be able to make new assessments related to this study in the course of this study. If no longer needed, we will destroy your data.

*Can we use your data for other research?*

Your data may also be important after this study for other medical research on suicidal thoughts and treatments. For this purpose, your child’s and your data will be stored at the sponsor site for 15 years. Please indicate in the consent form whether you agree to this. Do you not want to give your consent? Then you and your child can still take part in this study. Your child will receive the same treatment.

*What happens if there are accidental discoveries?*

It is possible that during the study we discover something that is important to your health or to the health of your family members. In that case, the investigator will contact your current therapist or GP or that of your child. You will then discuss what needs to be done with your doctor or therapist. By giving your consent on this form, you consent that your doctor or therapist or those of your child will be informed.

*Can you withdraw your consent for the use of your data?*

You can withdraw your consent for the use of your data at any time. Please note: if you withdraw your consent, and the investigators have already collected data for research, they are still allowed to use this information.

*Do you want to know more about your privacy?*

- Do you want to know more about your rights when processing personal data? Visit [Autoriteit Persoonsgegevens |](https://www.autoriteitpersoonsgegevens.nl/en).
- Do you have any questions about your rights? Or do you have a complaint about the processing of your personal data? Please contact the person who is responsible for processing your personal data. At present, this is:
  - AMC see Appendix A for contact details, and website.
- If you have any complaints about the processing of your personal data, we recommend that you first discuss this with the research team. You can also contact the Data Protection Officer of AMC. Or you can submit a complaint to the Dutch Data Protection Authority.

*Where can you find more information about the study?*

You can find more information about the study on the following website: <https://repairstudy.com>

1. **Will you receive compensation if you participate in the study?**

The tests and treatment for the study will not cost you anything. Your child will receive a voucher of €15,- per assessment. The four assessments for which your child will receive a voucher are the initial assessments, immediately after the treatment, and the ones 3 and 12 months after the treatment. Your child will receive €5.- for the online questionnaire 6 months after treatment. In total, your child can earn €65,- by participating in all 5 assessments. This does not apply to you, as parent(s)/caregiver(s). You receive €8 per assessment, which amounts to a maximum of €32,- in total. If you or your child stops before the study is finished, the compensation you and your child receive will be less.

1. **Are you insured during the study?**

Insurance has been taken out for everyone who participates in this study. The insurance pays for damage caused by the investigation. But not for all damage. In appendix B you will find more information about the insurance and the exceptions. It also states to who you can report the damage.

1. **We will inform your child’s GP and therapist**

The investigator will send your child’s GP and therapist a letter or email to let them know that you are taking part in the study. This is for the safety of your child. In the event of increasing suicidal thoughts or other crisis situations, we can contact the general practitioner and/or therapist of your child, for example, about the status of their current treatment.

1. **Do you have any questions?**

If you have any questions about this study, feel free to contact the research team. Would you like to get advice from someone who is independent from the study? In this case, you can contact Dr. Burger. He knows a lot about the study, but is not a part of this study. Do you have a complaint? Discuss it with the investigator or the doctor who is treating you. If you prefer not to do so, please visit the complaints officer or complaints committee of your hospital. Appendix A contains the contact information.

1. **How do you give consent for the study?**

You can first carefully think about this study. Then you tell the investigator of the treatment facility (independent of the health care provider) if you understand the information and if you want to take part or not. If you and your child want to take part, fill in the consent form that you can find with this information sheet. You, your child, and the investigator will get a signed version of this consent form.

Thank you for your time.

1. **Appendices to this information**

A. Contact information <*to be adjusted per participating centre*>

B. Information about the insurance

C. Overview of measurements

D. Consent form parent(s)/caregiver(s)

**Appendix A: contact information of AMC**

**Principal investigator:**

Professor Claudi Bockting

Meibergdreef 9

1105 AZ Amsterdam

Tel: +3120-8913600

E-mail: [c.l.bockting@amsterdamumc.nl](mailto:c.l.bockting@amsterdamumc.nl)

**Researchers**:

Dr. Nadia van der Spek

Meibergdreef 9

1105 AZ Amsterdam

Tel: +3120-8913600

E-mail: [n.vanderspek@amsterdamumc.nl](mailto:n.vanderspek@amsterdamumc.nl)

Professor Guy Bosmans

KU Leuven

Tiensestraat 102, 3000 Leuven, Belgium

Department of clinical psychology

Tel: +3216326187

E-mail: [guy.bosmans@kuleuven.be](mailto:guy.bosmans@kuleuven.be)

Luana Gavan

Meibergdreef 5

1105AZ Amsterdam

Tel: +3120-8913671

Email: [l.gavan@amsterdamumc.nl](mailto:s.sivananthan@amsterdamumc.nl)

Nele Bergers

KU Leuven

Tiensestraat 102, 3000 Leuven, Belgium

Department of clinical psychology

Tel: +3216326187

Email: [nele.bergers@kuleuven.be](mailto:nele.bergers@kuleuven.be)

**Independent doctor/expert:**

Dr. Huib Burger, MD

Universitair Medisch Centrum Groningen

Antonius Deusinglaan 1

9713 AV Groningen

Tel: +3150 361 6724

E-mail: [h.burger@umcg.nl](mailto:h.burger@umcg.nl)

**Complaints:**

Afdeling Patiëntenvoorlichting & Klachtenopvang, A0-404/406

Meibergdreef 5

1105 AZ Amsterdam

Tel: +3120-5663355

E-mail: [patientenvoorlichting@amc.nl](mailto:patientenvoorlichting@amc.nl)

**Data Protection Officer of the institution:**

E-mail: [privacy@amsterdamumc.nl](mailto:privacy@amsterdamumc.nl)

**For more information about your rights:**

Afdeling Patiëntenvoorlichting & Klachtenopvang, A0-404/406

Meibergdreef 5

1105 AZ Amsterdam

Tel: 020-5663355

E-mail: [patientenvoorlichting@amc.nl](mailto:patientenvoorlichting@amc.nl)

**Appendix B: Information about the insurance**

The AMC has taken out insurance for everyone participating in this study. The insurance covers damage caused by participation in the study. This applies to damage during the research or within four years after its end. You must report damage to the insurer within those four years.

The insurance does not cover all damage. At the bottom of this text, you will find a brief description of which damage is not covered.

These provisions are contained in the Decision on compulsory insurance for medical research involving human subjects. This decision can be found on www.ccmo.nl, the website of the Central Committee on research involving human subjects (see 'Library' and then 'Laws and regulations').

In the event of damage, you can contact the insurer [or claims representative] directly.

| The insurer of the research is:  Name insurer: Centramed B.A.  Adres insurer: Postbus 7374  2701 AJ Zoetermeer  Telephone: 070 301 70 70  E-mail: info@centramed.nl  Polisnumber: 624.528.303 |
| --- |

The insurance offers a cover of € 650.000 per test subject with a maximum of

€ 5.000.000 for the entire research and € 7.500.000 for damage resulting from medical research involving human subjects, that is reported per insurance year.

The insurance does not cover the following damage:

- damage due to a risk about which you have been informed in the written information. This does not
 apply if the risk is more serious than anticipated or if the risk was very unlikely;

- damage to your health that would also have occurred if you had not participated in the study;

- damage due to not (completely) following directions or instructions;

- damage to your descendants as a result of a negative effect of the research on you or your
 descendants;

- damage caused by an existing treatment method in research into existing treatment methods.

Furthermore, the subject is requested to contact Professor Claudi Bockting in this regard (see attachment A for contact information).

**Appendix C: Overview of assessments**

Assessments for the subject

| **Appointment** | **Time** | **Activities** | **Duration** | **Location** |
| --- | --- | --- | --- | --- |
| Eligibility screening | After intake | Interview | 15 minutes | At home/ online, or at your treatment facility |
| Measurement 1 | After eligibility screening and informed consent | Interview and questionnaires | 60 minutes | At home/ online, or at your treatment facility |
| Intervention | During first session, every two months, and last session | Questionnaire | 3 minutes per session | At home/ online, or at your treatment facility |
| Measurement 2 | After end of treatment | Interview and questionnaires | 60 minutes | At home/ online, or at your treatment facility |
| Measurement 3 | 3 months after treatment | Interview and questionnaires | 60 minutes | At home/ online, or at your treatment facility |
| Measurement 4 | 6 months after treatment | Questionnaires | 10 minutes | Online |
| Measurement 5 | 12 months after treatment | Interview and questionnaires | 60 minutes | At home/ online, or at your treatment facility |

Assessments for the parent(s)/caregiver(s)

| **Appointment** | **Time** | **Activities** | **Duration** | **Location** |
| --- | --- | --- | --- | --- |
| Measurement 1 | After eligibility screening of your child and informed consent | Questionnaires | 10 minutes | Online |
| Intervention | During first session, every two months, and last session | Questionnaire | 3 minutes per session | Online |
| Measurement 2 | After end of treatment | Questionnaires | 10 minutes | Online |
| Measurement 3 | 3 months after treatment | Questionnaires | 10 minutes | Online |
| Measurement 4 | 12 months after treatment | Questionnaires | 10 minutes | Online |

**Appendix D: Informed** **consent form - Parent(s)/caregiver(s)**

This consent form belongs to REPAIR study

I have been asked to give consent for my participation in this medical study for which my child also gave consent to participate:

Name of subject (child): …………………….. Date of birth: __/__/__

- I have read the information sheet. I was able to ask questions. My questions have been answered to satisfaction. I have had enough time to decide if I wanted to take part.
- I know that taking part is voluntary. I also know that at any time I can decide not to take part in the study. Or to stop taking part. I do not have to explain why.
- I give consent to collect and use my data. The investigators only do this to answer the question of this study.
- I know that some people will be able to see all of my data to review the study. These people are mentioned in this information sheet. I give consent to let them see my data for this review.
- Please tick yes or no in the table below.

| I give consent to store my data to use for other research, as stated in the information sheet. | Yes ☐ | No  ☐ |
| --- | --- | --- |
| I give consent to videotape our therapy sessions. | Yes ☐ | No  ☐ |
| I give consent to ask me after this study if I want to participate in a follow-up study. | Yes ☐ | No  ☐ |

- My child wants to take part in this study and I want to take part in this study.

Name parent/guardian: ………………………………

Signature: ………………………………… Date: __/__/__

Name other parent/guardian: …………………….

Signature: ……………………………………… Date: __/__/__

-----------------------------------------------------------------------------------------------------------------

I declare that I have fully informed the person(s) mentioned above about the study in question.

If any information becomes known during the study that could influence the parent’s/parents’/guardian’s/guardians’ consent, I will let them know in good time.

Name investigator (or their representative): …………………………

Signature: ……………………… Date: __/__/__

-----------------------------------------------------------------------------------------------------------------

<*if applicable*>

Additional information was given by:

Name: ………………………………………..

Job title: ………………………………………

Signature: ………………………………. Date: __/__/__

-----------------------------------------------------------------------------------------------------------------

* Delete what is not applicable.

*The parent(s)/guardian(s) will receive a complete information sheet and a signed version of the consent form.*

**Appendix C: Study Materials – Recruitment texts**

Recruitment text Young Adults – Belgium

**Onderzoek naar de behandeling voor suïcidaliteit onder jongvolwassenen en hun ouders**

**Wat willen we onderzoeken?**

Dit onderzoek richt zich op een behandeling van terugkerende donkere gedachten en pogingen tot zelfdoding bij jongvolwassenen, waarbij ook de ouders of andere verzorgers kunnen worden betrokken. In het REPAIR onderzoek wordt bekeken of deze therapie jongvolwassenen die veel last hebben van gedachten aan zelfdoding beter helpt. Deze therapie wordt boven op/aanvullend op de standaard behandelmogelijkheden aangeboden en we vergelijken deze met het ontvangen van enkel de standaardbehandeling.

**Wie kan mee doen?**

Als je wilt meedoen aan het onderzoek, moet je wel aan de volgende criteria voldoen:

- Je bent tussen de 18 en 25 jaar oud
- Je hebt vaak last van gedachten aan zelfdoding en/of je hebt recent een poging tot zelfdoding ondernomen
- Je hebt minstens één ouder en/of zorgfiguur die samen met jou wil meedoen aan de studie en de therapie

**Wat houdt deelname precies in?**

Als je mee doet aan het onderzoek, wordt door loting bepaald of je de aanvullende therapie krijgt, of de (reeds bestaande)/gebruikelijke behandeling. We vragen jou om mee te doen aan vier meetmomenten, waarbij je telkens een gesprek met een onderzoeker hebt over je klachten. Dit vindt plaats voor de behandeling, na de behandeling, na drie en 12 maanden, dit duurt ca. 45- 60 minuten per keer. Daarnaast vul je ook vragenlijsten in. Je ouders vragen we ook om vier keer vragenlijsten in te vullen, dit duurt ongeveer 10 minuten per keer.

**Wat kan deelname aan het onderzoek mij opleveren?**

Per meetmoment ontvang je een cadeaubon van €15, voor het eenmalig invullen van een korte vragenlijst ontvang je een cadeaubon ter waarde van €5. Ook je ouders krijgen voor elke meting een cadeaubon als dank, ter waarde van €8. Als je mee doet aan het onderzoek, blijf je gewoon in zorg op de plek waar dat je nu bent. Je krijgt altijd ook de standaardbehandeling aangeboden en op die manier krijg je de beste kwaliteit van hulp die we kunnen bieden.

Indien je interesse hebt om deel te nemen aan ons onderzoek, raden wij je aan om dit met je arts te bespreken.

Indien je graag wilt deelnemen aan ons onderzoek, aarzel dan niet om ons te contacteren op het volgende e-mailadres:

REPAIR studie:

[repairstudy@amsterdamumc.nl](mailto:repairstudy@amsterdamumc.nl)

***De Zelfmoordlijn in Nederland is altijd bereikbaar: via het gratis nummer 113 en/of via de chat*** [***www.113.nl/chatten***](http://www.113.nl/chatten) ***(24 uur beschikbaar).***

***De Zelfmoordlijn in België is altijd bereikbaar: via het gratis nummer 1813 (24/7 bereikbaar) of***[***online chat***](https://www.zelfmoord1813.be/chat-met-zelfmoordlijn-1813)***(elke dag van 18u30 tot 22u).***

Recruitment text Parent(s)/caregiver(s) – Belgium

**Onderzoek naar de behandeling voor suïcidaliteit onder jongvolwassenen en hun ouders**

Heeft uw jongvolwassen kind (18-25 jaar) last van gedachten aan zelfdoding, of heeft hij of zij een poging tot zelfdoding gedaan?

Wat willen we onderzoeken?

Dit onderzoek richt zich op een behandeling van terugkerende donkere gedachten en pogingen tot zelfdoding bij jongvolwassenen, waarbij ook de ouders of andere verzorgers kunnen worden betrokken. In het REPAIR onderzoek wordt bekeken of deze therapie jongvolwassenen die veel last hebben van gedachten aan zelfdoding beter helpt. Deze therapie wordt boven op/aanvullend op de standaard behandelmogelijkheden aangeboden en we vergelijken deze met het ontvangen van enkel de standaardbehandeling.

Wie kan mee doen?

U en uw kind kunnen meedoen aan het onderzoek, als aan de volgende criteria wordt voldaan:

- Uw kind is tussen de 18 en 25 jaar oud
- Uw kind heeft vaak last van gedachten aan zelfdoding en/of heeft recent een poging tot zelfdoding ondernomen
- U en/of een andere ouder of zorgfiguur is bereid om mee te doen aan de studie en de therapie

**Wat houdt deelname precies in?**

Als je mee doet aan het onderzoek, wordt door loting bepaald of uw kind de aanvullende therapie krijgt, of de (reeds bestaande)/gebruikelijke behandeling. We vragen uw kind om mee te doen aan vier meetmomenten, waarbij uw kind telkens een gesprek met een onderzoeker hebt over zijn/haar klachten. Dit vindt plaats voor de behandeling, na de behandeling, na drie en 12 maanden, dit duurt ca. 45- 60 minuten per keer. Daarnaast vult uw kind ook vragenlijsten in. U vragen we ook om vier keer vragenlijsten in te vullen, dit duurt ongeveer 10 minuten per keer.

**Wat kan deelname aan het onderzoek mij opleveren?**

Per meetmoment ontvangt je kind een cadeaubon van €15. Voor het eenmalig invullen van een korte vragenlijst ontvangt uw kind een cadeaubon ter waarde van €5. Ook u krijgt voor elke meting een cadeaubon als dank, ter waarde van €8. Als uw kind mee doet aan het onderzoek, blijft hij/zij gewoon in zorg op de plek waar dat hij/zij nu in behandeling is. Uw kind krijgt altijd ook de standaardbehandeling aangeboden en op die manier krijgt hij/zij de beste kwaliteit van hulp die we kunnen bieden.

Indien je interesse hebt om deel te nemen aan ons onderzoek, raden wij je aan om dit met je arts te bespreken.

Indien je graag wilt deelnemen aan ons onderzoek, aarzel dan niet om ons te contacteren op het volgende e-mailadres:

REPAIR studie:

repairstudy@amsterdamumc.nl

***De Zelfmoordlijn in Nederland is altijd bereikbaar: via het gratis nummer 113 en/of via de chat*** [***www.113.nl/chatten***](http://www.113.nl/chatten) ***(24 uur beschikbaar).***

***De Zelfmoordlijn in België is altijd bereikbaar: via het gratis nummer 1813 (24/7 bereikbaar) of***[***online chat***](https://www.zelfmoord1813.be/chat-met-zelfmoordlijn-1813)***(elke dag van 18u30 tot 22u).***

Recruitment text Young Adults – the Netherlands

**Onderzoek naar de behandeling voor suïcidaliteit onder jongvolwassenen en hun ouders**

Voel je je erg radeloos, waardeloos of heb je vaak het gevoel hebt dat je er beter maar helemaal niet meer kan zijn?

Dan is er voor jou en mogelijkheid om mee te doen aan het REPAIR-onderzoek.

**Wat willen we onderzoeken?**

Dit onderzoek richt zich op een nieuwe behandeling van terugkerende donkere gedachten en pogingen tot zelfdoding bij jongvolwassenen, waarbij ook de ouders of andere verzorgers worden betrokken. Deze therapie is in het buitenland positief ontvangen en in het REPAIR-onderzoek wordt bekeken of deze therapie inderdaad jongvolwassenen beter helpt die veel last hebben van gedachten aan zelfdoding. Deze nieuwe therapie wordt boven op de standaard behandelmogelijkheden aangeboden en we vergelijken die met het ontvangen van enkel de standaardbehandeling.

**Wie kan mee doen?**

Als je wilt meedoen aan het onderzoek, moet je wel aan de volgende criteria voldoen:

- Je bent tussen de 18 en 25 jaar oud
- Je hebt vaak last van gedachten aan zelfdoding en/of je hebt recent een poging tot zelfdoding ondernomen
- Je hebt minstens één ouder en/of zorgfiguur die samen met jou wil meedoen aan de studie en de therapie
- Je kampt niet met een langdurige en/of hevige verslaving
- Je hebt geen last van acute psychoses

**Wat houdt deelname precies in?**

Als je mee doet aan het onderzoek, wordt door loting bepaald of je de aanvullende therapie krijgt, of alleen de standaard behandeling. We vragen jou om mee te doen aan vier meetmomenten, waarbij je telkens een gesprek met een onderzoeker hebt over je klachten, Dit vindt plaats voor de behandeling, na de behandeling, na drie en 12 maanden, dit duurt ca. 45- 60 minuten per keer. Daarnaast vul je ook vragenlijsten in. Je ouders vragen we ook om vier keer vragenlijsten in te vullen, dit duurt ongeveer 10 minuten per keer.

**Wat kan deelname aan het onderzoek mij opleveren?**

Per deelname, ontvang je een cadeaubon van €15, voor het eenmalig invullen van een korter vragenlijst ontvang je een cadeaubon ter waarde van €5. In totaal kun je maximaal €65 aan cadeaubonnen ontvangen. Ook je ouders krijgen voor elke meting een cadeaubon als dank, ter waarde van €8, in totaal maximaal €32 aan cadeaubonnen. Als je mee doet aan het onderzoek, blijf je gewoon in zorg op de plek waar je dat nu bent. Je krijgt altijd ook de standaardbehandeling aangeboden en op die manier krijg je de beste kwaliteit van hulp die we kunnen bieden. Je behandelaar kan je hier meer over vertellen.

Indien je graag wilt deelnemen aan ons onderzoek, aarzel dan niet om ons te contacteren op het volgende e-mailadres:

Project REPAIR: [Contactgegevens]

***De Zelfmoordlijn in Nederland is altijd bereikbaar: via het gratis nummer 113 en/of via de chat*** [***www.113.nl/chatten***](http://www.113.nl/chatten) ***(24 uur beschikbaar).***

***De Zelfmoordlijn in België heeft is altijd bereikbaar: via het gratis nummer 1813 (24/7 bereikbaar) of online chat (elke dag van 18u30 tot 22u)***

Recruitment text Parent(s)/caregiver(s) – the Netherlands

**Wervingsfolder**

**Onderzoek naar de behandeling van suïcidaliteit onder jongvolwassenen en hun ouders**

Heeft uw jongvolwassen kind (18-25 jaar) last van gedachten aan zelfdoding, of heeft hij of zij een poging tot zelfdoding gedaan?

Dan is er een mogelijkheid voor u en uw kind, om mee te doen aan het REPAIR-onderzoek.

**Wat willen we onderzoeken?**

We onderzoeken een nieuwe behandeling van terugkerende donkere gedachten en pogingen tot zelfdoding bij jongvolwassenen, waarbij ook de ouders of andere verzorgers worden betrokken. Deze therapie is in het buitenland positief ontvangen en in het REPAIR-onderzoek kijken we of deze therapie een meerwaarde is voor jongvolwassenen die veel last hebben van gedachten aan zelfdoding. De nieuwe therapie wordt boven op de standaard behandelmogelijkheden aangeboden en we vergelijken die met het ontvangen van alleen de standaardbehandeling.

**Wie kan mee doen?**

U en uw kind kunnen meedoen aan het onderzoek, als aan de volgende criteria wordt voldaan:

- Uw kind is tussen de 18 en 25 jaar oud
- Uw kind heeft vaak last van gedachten aan zelfdoding en/of heeft recent een poging tot zelfdoding ondernomen
- U en/of een andere ouder of zorgfiguur is bereid om mee te doen aan de studie en de therapie
- Uw kind kampt niet met een langdurige en/of hevige verslaving
- Uw kind heeft geen last van acute psychoses

**Wat houdt deelname precies in?**

Als u mee doet aan het onderzoek, wordt door loting bepaald of uw kind de aanvullende therapie krijgt, of alleen de standaard behandeling. We vragen uw kind om mee te doen aan vier meetmomenten, waarbij hij/zij telkens een gesprek met een onderzoeker heeft over zijn of haar klachten. Dit vindt plaats voor de behandeling, na de behandeling, na drie en 12 maanden en duurt ca. 45-60 minuten per keer. Daarnaast vult u en/of de andere ouder of zorgfiguur ook vier keer vragenlijsten in, dit duurt ongeveer 10 minuten per keer.

**Wat kan deelname aan het onderzoek mij opleveren?**

Als dank voor uw deelname, ontvangen u en uw kind per meting voor het onderzoek, een cadeaubon. Uw kind ontvangen per meetmoment met de onderzoeker een cadeaubon van

€15 en daarnaast voor het eenmalig invullen van een korte vragenlijst €5. In totaal ontvangt uw kind voor deelname maximaal €65 aan cadeaubonnen. Iedere ouder ontvangt per meting een cadeaubon ter waarde van €8, in totaal maximaal €32 aan cadeaubonnen. Als uw kind mee doet aan het onderzoek, blijft hij of zij gewoon in zorg op de plek waar uw kind nu in behandeling is. Uw kind krijgt altijd ook de standaardbehandeling aangeboden en op die manier krijg je de beste kwaliteit van hulp die we kunnen bieden. De behandelaar van uw kind kan u hier meer over vertellen.

Indien je graag wilt deelnemen aan ons onderzoek, aarzel dan niet om ons te contacteren op het volgende e-mailadres:

Project REPAIR: [Contactgegevens]

***De Zelfmoordlijn in Nederland is altijd bereikbaar: via het gratis nummer 113 en/of via de chat*** [***www.113.nl/chatten***](http://www.113.nl/chatten) ***(24 uur beschikbaar).***

***De Zelfmoordlijn in België heeft is altijd bereikbaar: via het gratis nummer 1813 (24/7 bereikbaar) of online chat (elke dag van 18u30 tot 22u).***

1. In accordance with Article 29 of the Belgian Law related to experiments on humans (7 May 2004) [↑](#footnote-ref-1)
2. [↑](#footnote-ref-2)
3. These rights are guaranteed by the European Data Protection Regulation (GDPR), by the Belgian legislation on the protection of natural persons with regard to the processing of personal data and by the Law of 22 August 2002 on patient rights. [↑](#footnote-ref-3)
4. For clinical trials, the law requires this link with your records to be retained for 20 years. In the case of a advanced therapy medicinal product using human biological material, this period will be a minimum of 30 years and a maximum of 50 years in accordance with the Belgian Law of 19 December 2008 on the use of human biological material and the applicable royal decrees. [↑](#footnote-ref-4)
5. The database containing the results of the study will therefore not contain any combination of elements such as your initials, your gender and your full date of birth (dd/mm/yyyy). [↑](#footnote-ref-5)
6. The sponsor then undertakes to respect the constraints of the European General Data Protection Regulation (GDPR) and the Belgian legislation on the protection of natural persons with regard to the processing of personal data. [↑](#footnote-ref-6)
7. In accordance with Article 29 of the Belgian Law related to experiments on humans (7 May 2004) [↑](#footnote-ref-7)
8. In accordance with Article 29 of the Belgian Law related to experiments on humans (7 May 2004) [↑](#footnote-ref-8)
9. [↑](#footnote-ref-9)
10. These rights are guaranteed by the European Data Protection Regulation (GDPR), by the Belgian legislation on the protection of natural persons with regard to the processing of personal data and by the Law of 22 August 2002 on patient rights. [↑](#footnote-ref-10)
11. For clinical trials, the law requires this link with your records to be retained for 20 years.. [↑](#footnote-ref-11)
12. The database containing the results of the study will therefore not contain any combination of elements such as your initials, your gender and your full date of birth (dd/mm/yyyy). [↑](#footnote-ref-12)
13. The sponsor then undertakes to respect the constraints of the European General Data Protection Regulation (GDPR) and the Belgian legislation on the protection of natural persons with regard to the processing of personal data. [↑](#footnote-ref-13)
14. In accordance with Article 29 of the Belgian Law related to experiments on humans (7 May 2004) [↑](#footnote-ref-14)
